# Potential pitfalls of modelling ribosomal RNA data in phylogenetic tree reconstruction: Evidence from case studies in the Metazoa

**DOI:** 10.1186/1471-2148-11-146

**Published:** 2011-05-27

**Authors:** Harald O Letsch, Karl M Kjer

**Affiliations:** 1 Zoologisches Forschungsmuseum Alexander Koenig, Zentrum für molekulare Biodiversitätsforschung, Adenauerallee 160, 53113 Bonn, Germany; 2 Rutgers University, Department of Ecology Evolution and Natural Resources Faculty, 14 College Farm Rd., New Brunswick, NJ 08901, USA

## Abstract

**Background:**

Failure to account for covariation patterns in helical regions of ribosomal RNA (rRNA) genes has the potential to misdirect the estimation of the phylogenetic signal of the data. Furthermore, the extremes of length variation among taxa, combined with regional substitution rate variation can mislead the alignment of rRNA sequences and thus distort subsequent tree reconstructions. However, recent developments in phylogenetic methodology now allow a comprehensive integration of secondary structures in alignment and tree reconstruction analyses based on rRNA sequences, which has been shown to correct some of these problems. Here, we explore the potentials of RNA substitution models and the interactions of specific model setups with the inherent pattern of covariation in rRNA stems and substitution rate variation among loop regions.

**Results:**

We found an explicit impact of RNA substitution models on tree reconstruction analyses. The application of specific RNA models in tree reconstructions is hampered by interaction between the appropriate modelling of covarying sites in stem regions, and excessive homoplasy in some loop regions. RNA models often failed to recover reasonable trees when single-stranded regions are excessively homoplastic, because these regions contribute a greater proportion of the data when covarying sites are essentially downweighted. In this context, the RNA6A model outperformed all other models, including the more parametrized RNA7 and RNA16 models.

**Conclusions:**

Our results depict a trade-off between increased accuracy in estimation of interdependencies in helical regions with the risk of magnifying positions lacking phylogenetic signal. We can therefore conclude that caution is warranted when applying rRNA covariation models, and suggest that loop regions be independently screened for phylogenetic signal, and eliminated when they are indistinguishable from random noise. In addition to covariation and homoplasy, other factors, like non-stationarity of substitution rates and base compositional heterogeneity, can disrupt the signal of ribosomal RNA data. All these factors dictate sophisticated estimation of evolutionary pattern in rRNA data, just as other molecular data require similarly complicated (but different) corrections.

## Background

Progress of molecular techniques has eased the use of genomic data for phylogenetic analyses. Nevertheless, whole genomes are currently available for relatively few metazoans. Molecular studies of phylogenetic relationships within higher taxonomic groups, e.g. at the intra-ordinal level, therefore still rely on individual genes, among which the nuclear and mitochondrial ribosomal RNA genes are the most frequently sequenced. A pattern of highly variable positions, nested within conserved, slowly substituting sites across the alignment, yields a valuable resource for studying phylogenetic relationships of both recent and ancient splits [1-4]. This, combined with the ease of amplification, has lead to a widespread use of rRNA genes in phylogenetics and furthermore uncovered several specific properties of these genes, which should be considered, using these sequences as phylogenetic markers. Paired regions in rRNA sequences evolve via selectively neutral substitutions in the form of compensatory mutations [5] to maintain energetically stable secondary structures. Additionally, a strong bias of nucleotide composition between paired and unpaired areas has been observed [5,6]. Ribosomal RNA loop and stem regions are therefore subject to very different selectional regimes, which can hamper the use of rRNA genes for phylogenetic purposes [2,5,7-11]. In particular, correlated variation of nucleotides in stem regions, has been suggested to corrupt phylogenetic analyses as these covariation patterns of paired sites do not display independent phylogenetic signal. Ignoring this correlation results in an overestimation of phylogenetic information of these sites, which can lead to inflated measurements of tree robustness [12,13]. As a solution, rRNA secondary structural information as independent set of characters have been advocated to aid tree reconstruction by the use of specific RNA substitution models [12,14-17]. Application of RNA substitution models in phylogenetics is still confined to a few studies [11,18-27], most of them emphasing improvement of the analyses. In contrast, a recent study on hexapod phylogeny found mixed RNA/DNA model setups leading to a higher sensitivity to systematic problems ("long-branch attraction") [28]. This has been suggested as a result of potential homoplasy in loop positions. RNA models virtually downweight stem partitions, leading to an increased impact of loops. If these loop positions are saturated and/or misaligned, this "noisy" signal might dominate the phylogenetic signal of unsaturated stem positions and lead to inaccurate tree reconstruction.

**Figure 1:**
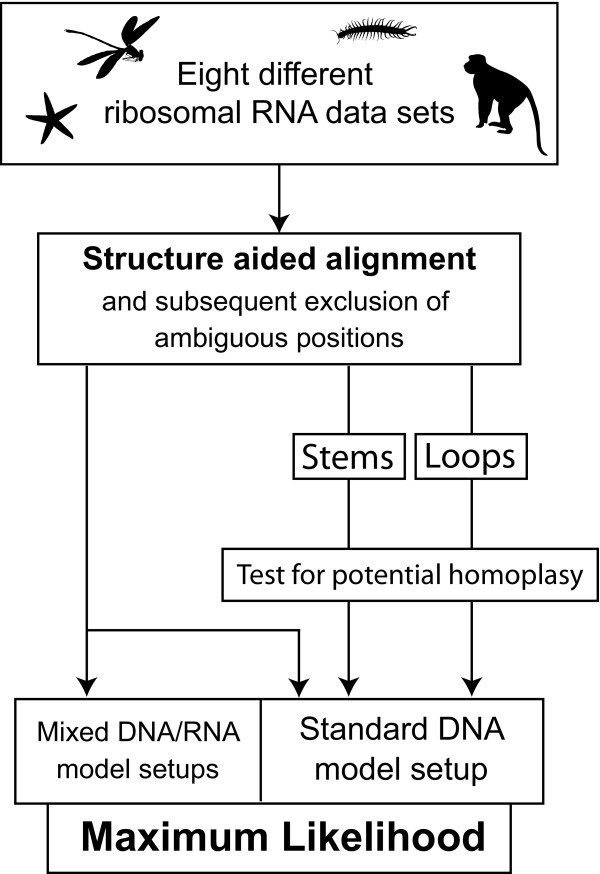
**Setup of the analyses**. Flowchart representing the setup of the analyses.

In the present study, we want to test this hypothesis by comparing the performance of mixed RNA/DNA model setups in the tree reconstruction of different ribosomal RNA data sets with the level of relative homoplasy in loop and stem positions. Current studies on the topic of modelling rRNA data in tree reconstruction have utilised simulation analyses [28,29], which can generally be seen to be a sophisticated complement to empirical studies in order to test hypotheses in algorithmically rooted phylogenetics. However, in Letsch et al. [28], tree reconstructions on simulated data were not able to reveal a potential correlation between homoplasy and data modelling. Consequently, the present analyses were based on case studies. Eight ribosomal RNA data sets were initially compiled, using from one to three mitochondrial and/or nuclear ribosomal RNA gene partitions, covering a broad spectrum of phylogenetic levels (Echinodermata (18S), Tunicata (18S), Heterobranchia (18S) Chilopoda (18S), Hexapoda (18S + 28S), Mammalia (12S + 16S), Primates (12S + 16S) and Anisoptera (12S + 16S + 28S)). All data sets were aligned with the RNASALSA alignment software [30], considering rRNA secondary structures. Ambiguously aligned positions where identified and excluded prior to the tree reconstruction. Based on the complete aligned data sets, we further conducted Maximum Likelihood (ML) tree reconstructions with the RAxML v7.2.6 software package [31-33] with (1) a standard DNA model setups and (2) 13 mixed RNA/DNA model setups. In the latter, loop positions are covered by a standard DNA model and stem positions are covered by a specific RNA model. Performance of different model setups was compared according to recent morphological and molecular expectations of taxonomy. To test the relative homoplasy between stem and loop regions, all alignments were divided into unpaired (loop) and paired (stem) positions according to a consensus secondary structure. Both partitions were then separately tested for homoplasy by estimating the level of substitutional saturation. Additionally, ML analyses were conducted on loop and stem partitions separately and the results were compared to the trees from the combined data sets. The analyses setup is depicted in Figure 1.

## Results

### Phylogenetic analyses

In the following, we represent and discuss the results of Echinodermata, Tunicata and Mammalia data sets as examples. Discussion on the results of all other data sets are provided in Additional file 1. To investigate the impact of homoplasy in loop regions on the behaviour of mixed RNA/DNA models setups in the tree reconstruction, the tree reconstruction results of all 13 RNA models were compared to the trees relying on the DNA model setup. Trees were evaluated in comparisons with recent morphological and molecular understanding of the accordant group, where we mainly focus on "benchmark clades" to check the reliability of each model setup. Clades were defined as "benchmark clades", if they have repeatedly received support in previous studies, based on independent morphological and/or molecular data.

#### Phylogeny of echinoderm classes

**Figure 2:**
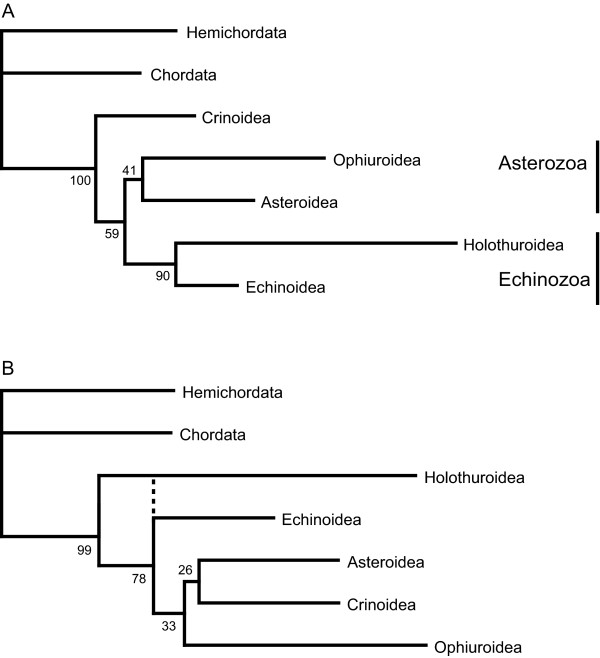
**Results Echinodermata**. Trees summarising the results of the tree reconstructions on Echinodermata. (A) Tree by the GTR and the RNA6A model setups, showing monophyletic Asterozoa and Echinozoa. Bootstrap (BS) values taken from the GTR model based tree reconstruction. (B) Results of all other mixed RNA/DNA model setups. Dotted line indicates alternative sistergroup relationship of Holothuroidea and Echinoidea. BS values taken from the RNA7A model based analyses.

Echinodermata is divided into five extant classes, the Crinoidea (sea lilies), Ophiuroidea (brittle stars), Asteroidea (starfishes), Holothuroidea (sea cucumbers) and Echinoidea (sea urchins). Monophyly in these five classes is well founded [34], whereas the relationships among them are still debated. Nevertheless, there is some consensus regarding major aspects of echinoderm phylogeny [34-36]. Crinoids are seen as the most basal split within Echinodermata, forming the sister group to the four remaining classes (Eleutherozoa). Furthermore, there is strong support for a sister group relationship of echinoids and holothurians (Echinozoa). Debates on the phylogenetic position of the stellate forms (starfishes and brittle stars) revolve around two competing hypotheses: are the ophiurids alone sister group to Echinozoa [37,38] or do asteroids and ophiuroids form a clade (Asterozoa), which is then the sister taxon to Echinozoa [34]? The above outlined hypotheses are only reflected by the results of the GTR and the RNA6A model setups. These trees all show basal Crinoidea and Eleutherozoa divided into Asterozoa and Echinozoa. In contrast, all other mixed RNA/DNA model setups show either Holothuroidea or a clade of Holothuroidea + Echinoidea the sister taxon to the rest of Echinodermata (Figure 2), thus clearly contradicting current expectations of echinoderm phylogeny.

#### The position of Appendicularia within Tunicata

**Figure 3:**
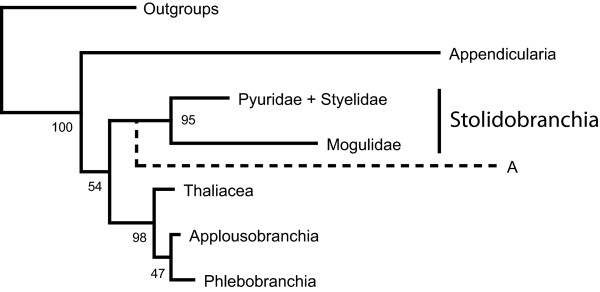
**Results Tunicata**. Tree summarising the results of the tree reconstructions based on Tunicata. The dotted line (A) indicates an alternative position of the Appendicularia. BS values taken from the RNA16A model based analyses.

Molecular approaches to the phylogeny of Tunicata are generally hampered by the base composition biases and elevated substitution rates in Aplousobranchia, Appendicularia and Mogulidae (Stolidobranchia). Appendicularia retain larval characters throughout their lifespan, which made an understanding of their phylogeny crucial for understanding the evolution of body plans and developmental modes in Tunicata [27]. Recent molecular studies using phylogenomic or rRNA data to target the phylogeny of Tunicata usually recover Appendicularia as sister group to all other tunicate groups [39-42]. However, this position is suspected to be a result of a "long branch attraction" artefact, due to genome-wide elevated substitution rates in this group [27,40]. As an alternative, Appendicularia as sister to Stolidobranchia has been recovered through analyses of 18S rRNA genes [27,39,43,44]. However, this position was generally weakly supported and has been discussed as a possible result of base composition bias in Appendicularia and Mogulidae, a family of Stolidobranchia [27]. These problems are reflected by the results of our study on tunicates (Figure 3), which either show Appendicularia as first split within Tunicata (RNA7C, E, F and RNA16A model setups) or as sister group to Stolidobranchia (all other model setups). According to the currently unresolved position of Appendicularia, none of these alternatives can be chosen as superior.

#### Relationships within Mammalia

**Figure 4:**
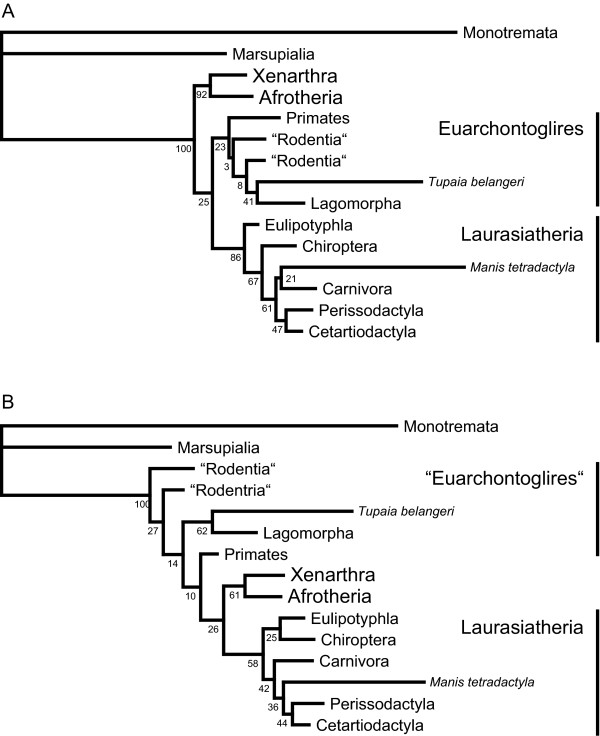
**Results Mammalia**. Trees summarising the results of the tree reconstructions on Mammalia. (A) Tree by the GTR and most mixed RNA/DNA model setups, showing the four monophyletic "superorders" Xenarthra, Afrotheria, Euarchontoglires and Laurasiatheria. BS values taken form the GTR model based analyses. (B) Tree derived from the RNA6A, RNA16 and RNA16B model setups, showing paraphyletic Rodentia as first splits within Eutheria and Xenarthra + Afrotheria as sistergroup to Laurasiatheria. BS values taken form the RNA16 model based analyses.

The first molecular analyses on the phylogeny of Mammalia, using mitochondrial genes have remarkably challenged previous morphological hypotheses on the relationship among mammalian groups [45]. However, subsequent studies on nuclear markers and more sophisticated analyses of mitochondrial genomes led to more consistent hypotheses of mammalian relationships, which are in several aspects congruent to morphological studies [45-51]. General congruence among these independent markers has resulted in a well resolved and strongly corroborated backbone tree of mammalian groups, representing the four "superorders" Xenarthra, Afrotheria, Euarchontoglires and Laurasiatheria and several subgroups, e.g. monophyletic Theria, the Paenungulata (containing elephants, hyraxes and sirenians), Tetytheria (elephants and sirenians), and Euarchonta (Scandentia + Dermoptera + Primates). Consequently, the evaluation of tree reconstructions targeting mammalian phylogeny has been formalised by defining theses groups as "benchmark clades" [52], whose appearance is used to evaluate the performance of the method that was used. In the present study, most analyses are highly congruent in their results of mammalian relationships and display many of the proposed benchmark clades with sufficient support. However, in the RNA6A, RNA16 and RNA16A analyses, a clade combining Afrotheria + Xenarthra is sistergroup to Laurasiatheria and Rodentia appear paraphyletic to the remaining eutherian groups, with Muroidea + *Anomalurus *as first split within Eutheria. This reflects the suggestions of several previous analyses based on mt genes, but must been interpreted as a result of model misspecification ignoring among-site rate variation [53,54] and compositional bias [55]. In contrast, all other analyses show Afrotheria + Xenarthra as first split within Eutheria and paraphyletic Rodentia, but the latter are nested within monophyletic Laurasiatheria. It is notable, that bootstrap support values for potentially correct groupings increased, if mixed RNA/DNA model setups are applied (cf. Figure 4 and Additional file 2 for complete mammalian trees).

### Separate analyses of loops and stems

**Figure 5:**
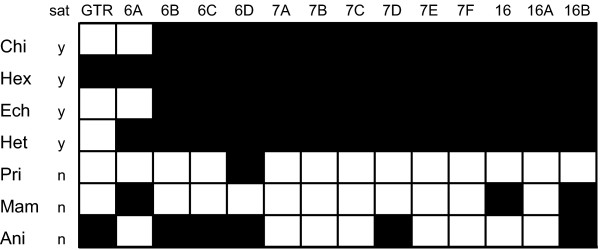
**Summary of all tree reconstruction results**. The matrix summarises all results of the tree reconstructions in context of substitution saturation (sat: y = yes, n = no). White boxes indicate a potentially correct tree hypotheses, whereas black boxes indicate probably wrong tree hypotheses. The results of the tunicate data set are not shown, as they could not been construed unequivocally. Abbreviations: Chilopoda: Chi, Hexapoda: Hex, Echinodermata: Ech, Tunicata: Tun: Heterobranchia: Het, Primates: Pri, Mammalia: Mam, Anisoptera: Ani.

Homoplasy due to multiple substitutions was tested with the index of substitution saturation (ISS) [56,57], which assumes a critical index of substitution saturation (ISSc) that defines a threshold for significant saturation in the data. The ISSc is compared with the observed ISS of the data. If the ISS value is larger than the critical ISSc values, saturation is assumed. To contribute to different tree shapes, the ISSc is estimated, using a symmetrical (balanced) and an asymmetrical (pectinate) tree topology. The test for homoplasy reveals striking differences between paired and unpaired positions. In the stem portions of all data sets, the asymmetrical and the symmetrical ISSc is always larger than the observed ISS. The differences are significant, thus indicating that the paired partitions are not saturated. In contrast, we detected potential saturation of substitution in the unpaired positions of several data sets. For Echinodermata, Hexapoda, Tunicata, Chilopoda and Heterobranchia, the ISS of was notably larger than the asymmetrical ISSc, suggesting substantial saturation in these alignments. The complete results of the saturation tests are summarised in Table 1. The comparison matrix in Figure 5 further depicts the results of all tree reconstruction analyses in relation of substitution saturation in the loop partitions. The saturation test results of the loop and stem partitions were additionally compared to the saturation test results of the combined data sets of the groups exhibiting saturation in the loop regions. As displayed in Table 2 saturation vanishes in all of the combined data sets.

**Table 1:** Data set characteristics

Taxon	Gene(s)	Species	Alignment length*	Partition	Saturatio Iss n	Iss.c S	P	Iss.c A	P
Chilopoda	18S	61	2576 (1822)	stems	0.062	0.715	0.000	0.398	0.000
				loops	0.806	0.710	0.426	0.390	0.001
Hexapoda	18S+28S	94	9217 (4413)	stems	0.181	0.765	0.000	0.476	0.000
				loops	0.549	0.758	0.000	0.465	0.101
Echinodermata	18S	144	2045 (1706)	stems	0.129	0.721	0.000	0.406	0.000
				loops	0.457	0.722	0.000	0.408	0.418
Heterobranchia	28S	50	3609 (2388)	stems	0.033	0.652	0.000	0.302	0.000
				loops	0.473	0.649	0.491	0.297	0.492
Tunicata	18S	88	1990 (1960)	stems	0.239	0.729	0.000	0.419	0.000
				loops	0.444	0.741	0.000	0.438	0.898
Primates	12S+16S	54	1788 (1362)	stems	0.115	0.743	0.000	0.441	0.000
				loops	0.327	0.762	0.000	0.471	0.000
Mammalia	12S+16S	126	3102 (1875)	stems	0.168	0.775	0.000	0.492	0.000
				loops	0.393	0.764	0.000	0.474	0.027
Anisoptera	12S+16S+28S	108	5968 (5239)	stems	0.043	0.756	0.000	0.460	0.000
				loops	0.059	0.733	0.000	0.425	0.000

Characteristics of the applied test data sets including the results of the test for substitution saturation in loop and stem partitions. Iss: estimated index of substitution saturation for the data set. Iss.c S and Iss.c A: critical values for the index of substitution saturation (ISS) if the true tree is symmetrical (S) or asymmetrical (A). Iss > Iss.c indicates saturation.

*original (ambiguous positions excluded)

**Table 2:** ISS test for combined data sets

Subgroups	Saturation test				
	Iss	Iss.c S	P	Iss.c A	P
Chilopoda	0.314	0.760	0.000	0.467	0.002
Hexapoda	0.392	0.799	0.000	0.533	0.000
Echinodermata	0.484	0.769	0.000	0.482	0.961
Heterobranchia	0.542	0.797	0.000	0.531	0.572
Tunicata	0.264	0.782	0.000	0.503	0.000

Results of the index of substitution saturation test for combined data sets, where homoplasy due to multiple substitution had been previously been detected in the loop partitions. Iss.c S and Iss.c A: critical values for the index of substitution saturation (ISS) if the true tree is symmetrical (S) or asymmetrical (A). Iss > Iss.c indicates saturation.

**Figure 6:**
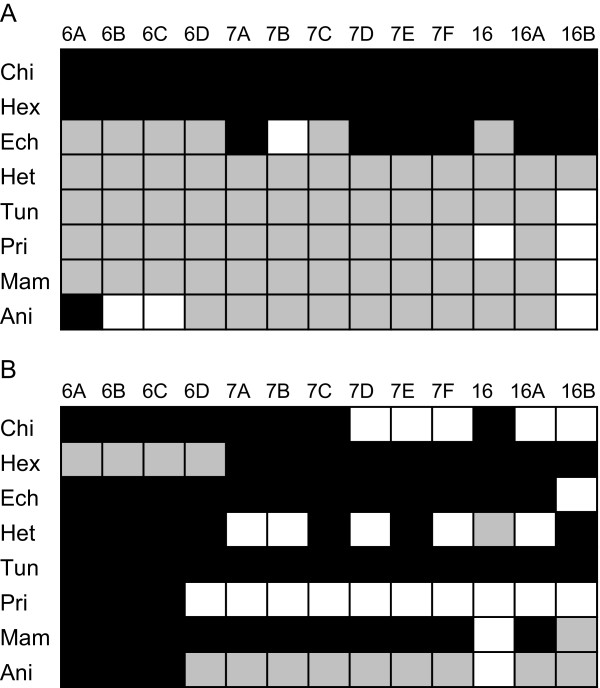
**Summary of Robinson-Foulds distances**. Robinson-Foulds distance scores between the complete data sets and the data sets restricted to (A) unpaired positions and (B) paired positions. Black fields indicate an increased RF distance between the combined data sets and the restricted data sets, under application of a mixed model setup on the combined data set. Grey fields indicate an decreased RF distance and white fields indicate no RF distance between combined data sets and the restricted data sets. Abbreviations correspond to Figure 5.

Subsequently, ML tree reconstructions were conducted on the separated loop and stem partitions, using a DNA model setup. To characterise the phylogenetic signal in both partitions, we checked whether the trees from the paired or the unpaired partition were more congruent to the combined data results. Trees resulting from all three setups (combined, paired, unpaired) were compared with the Robinson-Foulds [58] (RF) distance score, which accounts for topology differences. This indicates a closer similarity of trees based on combined and unpaired data. Comparisons between the combined data set, analysed under different mixed model schemes, usually strengthen this effect. With the exception of Chilopda and Hexapoda, most RF distances between the combined data and the unpaired data diminished, whereas RF distances between the combined data and the paired data often increased (cf. Figure 6 and Additional file 3 Table S4 and S5).

## Discussion

### Relative homoplasy and RNA modelling

To our knowledge, this is the first work on a separate characterization of homoplasy in paired and unpaired regions of rRNA sequences. We examined relative homoplasy separately, as due to their distinct physiological function in protein biosynthesis, paired and unpaired positions can be expected to evolve differentially and might therefore also differ in their sensitivity to numerous substitutions occurring at the same position, thus hiding or completely erasing phylogenetic signal. Our results indicate separate exploratory analyses of loops and stems as crucial, because homoplasy due to multiple substitutions in loop positions could not be detected if the combined data sets (loops + stems) are tested for excessive homoplasy (Table 2). Pooling loop portions may be subject to the same kind of underestimation of homoplasy, if rates among loop regions are highly heterogeneous, as is apparent in Van de Peer et al. [59]. Thus, it may be advisable to estimate homoplasy in each loop region separately. These homoplastic substitution patterns have generally been addressed as "substitutional saturation" [60,61]. However, "saturation" is a concept that is only relevant to distance based analyses, where "saturation" refers to saturation curves, in which increasing phylogenetic depth does not increase pairwise distances. Phylogenies have nodes at many levels, from the tips to the root, and character based analyses can insulate homoplasy and mediate errors due to homoplastic sites in ways that distance based analyses cannot [62,63], for example by increasing the taxon sampling to break up long branches [64-69]. In this context, it can further be stated that "saturation" is not an inherent character of an aligned sequence position in a given alignment, it rather depends on the considered phylogenetic level. Additional measurements of the ISS in the tunicate data set, applied to the loop partitions of distinct monophyletic subgroups within Tunicata, shows an ISS significantly smaller than the ISSc for both symmetrical and asymmetrical topologies, thus indicating a decrease of homoplasy in these groups (Table 3). The ISS method applied here accounts for position specific nucleotide frequency pattern in a given alignment, which are supposed to reflect the occurrence of multiple substitutions in the data set [57], thus implying relative homoplasy. Therefore, the use of the ISS might be a reasonable heuristic to estimate the level of homoplasy according to the deep splits discussed in the considered data sets.

**Table 3:** ISS test for several subgroups within Tunicata

Subgroups	Saturation test				
	Iss	Iss.c S	P	Iss.c A	P
Phlebobranchia	0.584	0.747	0.031	0.536	0.524
+ Aplousobranchia					
Phlebobranchia	0.533	0.755	0.001	0.556	0.726
Thaliacea	0.807	0.760	0.390	0.626	0.001
Mogulidae	0.251	0.752	0.000	0.593	0.000
Stylidae + Pyuridae	0.330	0.746	0.000	0.481	0.014
Stylidae	0.183	0.748	0.000	0.569	0.000

Results of the index of substitution saturation test for different tunicate subgroups. Iss.c S and Iss.c A: critical values for the index of substitution saturation (ISS) if the true tree is symmetrical (S) or asymmetrical (A). Iss > Iss.c indicates saturation.

As depicted in Figure 5, most of the RNA models only lead to reasonable tree hypotheses, if the loop regions are found to contain meaningful phylogenetic data. In data sets identified as saturated, nearly all RNA models failed to recover an expected hypothesis. In contrast, the standard DNA model setup usually led to trees congruent with recent views on the relationships in the accordant groups. In this context, especially the results of the hexapod data are interesting, as these analyses did not provide superior trees by the DNA models setups, although saturation is detected. In the case of hexapods, no model setup led to the expected tree hypothesis. This indicates a generally insufficient phylogenetic signal of the 18S and 28S RNA data to resolve the shortest of the internodes among the deep hexapod splits. However, in [28], Bayesian inference analyses of the identical data set led to wrong tree hypotheses in the mixed RNA/DNA model setup, but not in the standard DNA model setup. Kjer [11] found that topologies were virtually identical between the standard and RNA models, but support values varied: in some cases, expected nodes were more strongly supported with GTR models (Archaeognatha, Pterygota, Paraneoptera + Holometabola, and Hymenoptera) other cases favoured the doublet model (Hexapoda, lestoids sister to other Zygoptera). Similarly, support for a paraphyletic Anisoptera (presumably incorrect) went down with the doublet model. Nevertheless it is noteworthy, that mixed RNA/DNA model setups frequently led to an increased support for probably correct clades in data sets without homoplastic loops (Mammalia and Primates), thus supporting previous studies on RNA models in phylogenetics [14,19,23].

Our analyses setup also allows comparisons between the different RNA model setups. Current RNA substitution models can be divided into two distinct classes, rooted in population genetics [5,10]. Models of the first class assume a one-step process of compensatory substitution in paired positions, thus allowing double substitutions (e.g. AU ↔ GC): a mutation in a base pair (AU → GU) may led to slightly deleterious UG or GU pairs. If selection against these intermediates is strong, these are kept in low frequency in the population. If a second mutation occurs at the corresponding site (GU → GC), drift in gene frequency may lead to a domination of this new base pairing in the population (RNA6A-D, RNA7A-B, D and RNA16A). In contrast, models of the second class assume a two-step process of compensatory substitution in paired positions, considering one substitution in base-pairs, with a probability of zero for all double substitutions. This approach considers a fixation of intermediate states in the population at a high frequency, after a mutation in a base pair and before a second mutation at the corresponding site (RNA7C, F and RNA16, B). RNA models can be further discriminated by their treatment of mismatches: 6-state models completely ignore these pairings, 7-state models lump all mismatches in one category, whereas 16-state models apply distinct frequency and substitution rate parameters to the individual mismatches.

Previous studies on RNA models in phylogenetics have predicted the superiority of models allowing double substitutions and the superiority of the most general models (RNA6A, RNA7A) [5,10,70]. The latter is corroborated by the AICc modeltest of the present analyses, which frequently show higher likelihoods and AICc values for the most general models (see Additional file 3 Table S2 and S3). Furthermore, the RNA6A model led to the expected topologies in two data sets (Echinodermata and Chilopoda) showing relative homoplasy in loop partitions, whereas all other RNA models fail to display presumably correct trees, if significant homoplasy was identified (Figure 5). In this context, the RNA6D and RNA16B models are performing worst, as both are only able to display one potentially correct tree hypotheses. Additionally, congruencies between the performance of RNA models and the results of the AICc modeltest can be drawn from our results. According to the AICc modeltest, in the class of the RNA6 models, the most general RNA6A model is always superior to all other RNA6 models (see Additional file 3 Table S2 and S3), which is further congruent to its performance in tree reconstruction analyses. This is not reflected by the RNA7 and RNA16 models, where the models with the highest AICc scores (RN7A-B and RNA16 did not perform best (cf. Figure 5).

### Potential pitfalls of RNA modelling

Consequences of different evolutionary constraints in stem and loop regions of rRNA sequences for phylogenetic analyses has long been suspected and led to different recommendations for weighting stem positions in parsimony analyses [2,7,8]. Beside suggestions for simple one-half weighting of paired positions [7], empirical investigation of compensatory substitution rates in stem positions [8] reveals a rate of about 40% of that expected under a hypothesis of perfect compensation. Therefore, the weighting of stem characters is suggested to be reduced by no more than 20%. Consequently, in model based tree reconstruction methods, like Bayesian inference and Maximum Likelihood, it should be reasonable to use specific RNA models (which can be seen as an algorithmic equivalent to weighting stem positions in Maximum Parsimony) as simply applying a standard DNA model to data from one part of the helical regions. Application of these RNA models has frequently been justified by a consistent phylogenetic signal of coevolved paired sites, decreasing the information content in the data [5,18]. Analyses ignoring this interdependence should tend to overestimate the support for dubious or even wrong nodes in a tree [13]. Due to a reduced number of effective sites, the application of specific RNA models, which take interdependencies into account, reduces tree confidence, but is more reliable in the light of the information content in the data [12,16].

Our results actually imply a reduced impact of stem positions in the combined data set, if mixed RNA/DNA model setup are used. This is depicted by the tree distance results of the separate analyses of the stem and loop partitions. In most data sets, the distances between the trees based on only the loop partition and the combined data are reduced, if RNA models are applied for the combined data, whereas the distances between the stem partition and the combined data are mostly enlarged (Figure 2). This could have been expected, if coevolution in paired sites is assumed and thus these positions do not provide independent phylogenetic information. However, for several of the currently tested data sets, the substitution saturation test reveals that the unpaired positions clearly experience excessive homoplasy, which indicates a loss of phylogenetic information, as these positions are no longer informative [71]. In this context, stem positions in the current data sets should contain more reliable signal, compared to loop regions, as they exhibit a much lesser grade of homoplasy due to multiple substitutions. Consequently, the application of RNA models increases the relative impact of noisy positions in the data set and reduces the influence of more informative portions. Thus, the results of the current analyses corroborate the hypothesis proposed by Letsch et al. [28].

RNA models doubtlessly provide a better depiction of the phylogenetic information content of rRNA data sets, but this might be a trap, if homoplasy is far greater in loop positions. In this case, the informative phylogenetic content is obscured by noise. The situation might probably be depicted best, if we thought of a weighting scheme for rRNA data sets: in standard DNA model setup, the signal of paired positions is virtually weighted twice, as both positions are linked and signal of pairs can be seen redundant. As outlined above, previous studies have mostly targeted this as a problem [5,10,12,13,18,19], but the current analyses showed relative homoplasy as delimiting the confidence of the phylogenetic signal provided by loop regions, revealing a more or less hidden coherence between two factors - covariation and homoplasy - contributing to the phylogenetic signal of the rRNA data sets. For this scenario it can be stated, that in contrast to a previously proposed overestimation of wrong support by ignoring site interdependencies [13], the application of RNA models will tend to overestimate the support for dubious or wrong nodes in a tree.

As depicted above, loops and stems can be expected to experience different selectional regimes, which has resulted in the development of the specific RNA models. Nevertheless, it has been noted as early as 1991 [72] that substitution rates do not fit neatly into stem-loop partitions, and thus weighting according to stems vs. loops might be problematic, which was later demonstrated by Van de Peer [59]. Consequently, selectional constraints on rRNA may not only differ between paired and unpaired regions, but also among the individual loop or stem regions, which would depend on the individual function and their relative location in the 3D rRNA molecule. Binding sites of ribosomal proteins, for example protein L11-binding domain (L11-BD) within the LSU rRNA domain II and the sarcin-ricin loop within domain VI, constituting the GTPase-associated center [73] or the LSU rRNA domain V, which contains the peptidyl transferase center (PTC) [74], are highly conserved throughout metazoa. Furthermore, many of the rRNA regions of domain IV that are involved in tRNA and inter-subunit interactions are also preserved [74,75]. In contrast, the domain I of the mt LSU is highly variable on sequence level and until now, no conserved secondary structures could be detected [4,73]. Consequently, the partitioning into loops and stems must be seen as only an relative coarse approximation to model rRNA sequences. In future phylogenetic studies on rRNA, more sophisticated partitioning schemes, depending on the function, base composition and relative location of rRNA regions, would be able to enhance model based tree reconstruction analyses

## Conclusions

The results of the present study can be interpreted as a trade-off between using specific RNA models for a hopefully more accurate estimation of covariation in paired sites and the risk of augmenting relatively homoplastic unpaired positions in the tree reconstruction. For future phylogenetic studies based on rRNA sequences, we would therefore highly recommend a separate test for saturation of substitution in loop and stem partitions of the aligned data set. The use of a mixed RNA/DNA model setup should be avoided if saturation occurs in the loop partitions, as otherwise the valuable phylogenetic signal of the stem partitions might be masked by potentially noisy signal provided by the loops. In contrast, if no substantial homoplasy is detected in the data, the use of mixed RNA/DNA models can be highly recommend, as these lead to an increased support to probably correct clades.

Based on the presents results, we cannot advocate an general exclusion of the potentially noisy loop positions: First, noise is not an inherent character of a certain nucleotide position, but depends on the considered phylogenetic level. And second, differences *among *loop (or stem) regions can be expected and excluding these regions as a whole reduces the phylogenetic signal of the data set. Consequently, we rather recommend to think about enhanced partitioning strategies, which would allow a more careful modelling of rRNA sequences and provide a first approach to detect noisy signal *among *loop (or stem) partitions.

However, covariation and substitution saturation are only two parameters of the evolutionary inherent pattern displayed (or hidden) in the data. Other phenomena, like non-stationarity of substitution rates across sites and branches as well as base composition heterogeneity, might also maul the signal content of the data set. A previous study [26] based on nuclear rRNA genes, identified deviation of base composition in certain clades as probably misleading tree reconstruction analyses, rather than the covariation pattern in stem regions. A sophisticated estimation of evolutionary pattern in rRNA sequence data is therefore principally desirable and newly developed methods should be applied, which are able to consider background knowledge as covariation, non-stationary processes or heterogeneity in the data [26,76].

## Methods

### Compilation of data sets

As exemplary data sets to test the performance of we chose Echinodermata (18S), Tunicata (18S), Heterobranchia (18S) Chilopoda (18S), Hexapoda (18S + 28S), Mammalia (12S + 16S), Primates (12S + 16S) and Anisoptera (12S + 16S + 28S). All sequences were downloaded from NCBI Genbank (Additional file 4 provides complete taxon tables, including Genbank accession numbers). To apply mixed RNA/DNA models in the tree reconstruction, we had to infer reliable individual secondary structures. Consequently, we only considered 18S sequences with at least 1700 bp and 28S sequences with at least 3000 bp. 12S and 16S rRNA sequences in the primate and mammalian data sets were taken from entire mitochondrial genomes and therefore span the entire rRNA locus. In Anisoptera, the 12S and 16S rRNA sequences have minimum lengths of 500 bp and 1250 bp respectively. For the combined data sets, we only considered taxa that were represented by all genes.

### Alignment procedures

Alignment was done with the RNASALSA software [30], which aligns ribosomal RNA sequences by utilising existing hypotheses of structural patterns, in order to constrain thermodynamic folding algorithms and favour the alignment of sites that contain compensatory substitutions. In three steps, RNASALSA accumulates structure information, until each sequence receives its individual secondary structure string. In the first steps, conserved structure features are recognized via primary sequence conservation and consistent and/or compensatory substitution, which provides a structure skeleton for the next step, where the more variable regions gain structures by thermodynamic folding. Finally, the combined sequence and structure strings are simultaneously aligned, where sequence and structure information come into account. The program uses structural constraints as an input file, and our constraints of nuclear and mitochondrial SSU/LSU genes (see Supplement S1), were originally retrieved from the European Ribosomal Database (ERDB) [77-79]. The structures of these sources are coded in the proprietary DCSE format and were recoded into the required dot-bracket format with the program *extractfromdcse *of the PHASE software package [18,80]. The ERDB homepage is offline now, but readily recoded constraint structure files (representing various metazoan groups), as well as tools to divide loop and stem partitions, are available at the RNAsalsa homepage http://rnasalsa.zfmk.de. RNASALSA also requires a "pre-alignment" input file, which was obtained from the E-INS-i algorithm of the MAFFT alignment package [81], using default settings. The stringency settings for adoption of secondary structures in different alignment steps was relaxed (0.51), as we wanted to retain as much structure information as possible (see [30] for a detailed description of the RNASALSA method). Subsequent evaluation of the alignments was done with ALISCORE[82], which identifies ambiguously aligned regions in multiple sequence alignments. For gap treatment (g), window size (ws) and random pairwise comparisons (pc), the following settings were used (g: gaps as ambiguous characters; ws: four positions; pc: taxa^2^).

### Maximum Likelihood analyses

Maximum Likelihood analyses were conducted with RAxML v7.2.6 [31-33], which is an enhanced program for computing phylogenetic trees based on Maximum likelihood inference that includes RNA substitution models (RNA6A-D, RNA7A-F, RNA16, RNA16A and RNA16B, for a detailed description of the RNA models, please refer to the manual of the PHASE software package [80]). To define paired an unpaired partitions, the consensus structures in dot bracket format were used, obtained from the RNAsalsa alignments. In the standard DNA setup, the GTR model was used with all model parameters estimated from the data, with among site rate variation modelled with gamma distributed rates across sites with four discrete rate categories. Additionally, model parameters were optimised for different partitions, representing SSU and LSU rRNA sequences respectively. In the RNA model setups, a third partition is defined, according to the consensus secondary structure of the whole alignment and all paired position are extracted and pooled in third partition. The consensus structure provided by RNAsalsa Model fitting in both single nucleotide partitions is applied as in the standard model setup and the in paired nucleotide partition a specific RNA model is used. Within each class of RNA-models, the best model is evaluated by an Akaike Information criterion (AICc) test.

### Test for homoplasy

Relative homoplasy was examined between loop and stem regions. For this purpose, the aligned data sets were divided into paired and unpaired partitions, according to the consensus structures, provided by the RNASALSA alignments. Subsequently, each partitions was compared for the level of homoplasy in the data, using the substitution saturation test of the program package DAMBE v5.2.9 [56,57], which estimates an "index of substitution saturation", based on the notion of entropy in information theory. Prior to the saturation test, we accounted for invariant sites, which provides a more reasonable estimation of potential saturation in the data sets [57].

## Authors' contributions

HL designed the analyses and wrote the paper with comments and revisions from KMK.

## Supplementary Material

Additional file 1 **Additional tree reconstruction results**. Detailed discussion on the results of tree reconstruction of Chilopoda, Hexapoda, Anisoptera, Primates and Heterobranchia. Click here for file

Additional file 2 **Tree reconstruction results**. All trees (Newick le format) provided by the DNA model setups the and mixed RNA/DNA model setups of all applied data sets. Click here for file

Additional file 3 **Tables**. Tables providing the Genbank accession numbers of constraint sequences used for the RNASALSA alignment, the detailed results of the AICc test and the detailed results of the Robinson-Foulds distance measurements. Click here for file

Additional file 4 **Taxa list**. A list of all applied sequence data with according Genbank accession numbers. Click here for file

## References

[B1] Woese C Bacterial Evolution Microbiological Reviews 1987 51 2 221 271 [ISI:A1987H609200004] 2439888 10.1128/mr.51.2.221-271.1987 PMC373105

[B2] Hillis D Dixon M Ribosomal DNA: molecular evolution and phylogenetic inference Q Rev Biol 1991 66 4 411 453 [PM:1784710] 10.1086/417338 1784710

[B3] Gillespie J Characterizing regions of ambiguous alignment caused by the expansion and contraction of hairpin-stem loops in ribosomal RNA molecules Mol Phylogenet Evol 2004 33 3 936 943 [PM:15522814] 10.1016/j.ympev.2004.08.004 15522814

[B4] Gillespie J Johnston J Cannone J Gutell R Characteristics of the nuclear (18S, 5.8S, 28S and 5S) and mitochondrial (12S and 16S) rRNA genes of Apis mellifera (Insecta : Hymenoptera): structure, organization, and retrotransposable elements Insect Mol Biol 2006 15 5 657 686 [ISI:000241625100013] 10.1111/j.1365-2583.2006.00689.x 17069639 PMC2048585

[B5] Higgs P RNA secondary structure: physical and computational aspects Quarterly Reviews of Biophysics 2000 33 3 199 253 [ISI:000168335500001] 10.1017/S0033583500003620 11191843

[B6] Gutell R Cannone J Konings D Gautheret D Predicting U-turns in ribosomal RNA with comparative sequence analysis J Mol Biol 2000 300 4 791 803 [ISI:000088508500010] 10.1006/jmbi.2000.3900 10891269

[B7] Wheeler W Honeycutt R Paired sequence difference in ribosomal RNAs: evolutionary and phylogenetic implications Mol Biol Evol 1988 5 90 96 [PM:3357414] 3357414 10.1093/oxfordjournals.molbev.a040480

[B8] Dixon MT Hillis DM Ribosomal RNA secondary structure: compensatory mutations and implications for phylogenetic analysis Mol Biol Evol 1993 10 256 267 8450759 10.1093/oxfordjournals.molbev.a039998

[B9] Kjer K Baldridge G Fallon A Mosquito Large Subunit Ribosomal-Rna - Simultaneous Alignment of Primary and Secondary Structure Biochimica et Biophysica Acta - Gene Structure and Expression 1994 1217 2 147 155 [ISI:A1994MZ65700004] 10.1016/0167-4781(94)90028-0 8110829

[B10] Savill N Hoyle D Higgs P RNA sequence evolution with secondary structure constraints: Comparison of substitution rate models using maximum-likelihood methods Genetics 2001 157 399 411 [ISI:000166359400035] 11139520 10.1093/genetics/157.1.399 PMC1461489

[B11] Kjer K Aligned 18S and insect phylogeny Syst Biol 2004 53 3 506 514 [ISI:000222351000010] 10.1080/10635150490445922 15503677

[B12] Tillier E Collins R Neighbor Joining and Maximum-Likelihood with Rna Sequences - Addressing the Interdependence of Sites Mol Biol Evol 1995 12 7 15 [ISI:A1995QA17400002]

[B13] Galtier N Sampling properties of the bootstrap support in molecular phylogeny: influence of nonindependence among sites Syst Biol 2004 53 38 46 10.1080/10635150490264680 14965899

[B14] Schoeniger M von Haeseler A A stochastic model for the evolution of autocorrelated DNA sequences Mol Phylogenet Evol 1994 3 3 240 247 [PM:7529616] 10.1006/mpev.1994.1026 7529616

[B15] Rzhetsky A Estimating substitution rates in ribosomal RNA genes Genetics 1995 141 2 771 783 [PM:8647409] 8647409 10.1093/genetics/141.2.771 PMC1206772

[B16] Tillier E Collins R High apparent rate of simultaneous compensatory base-pair substitutions in ribosomal RNA Genetics 1998 148 4 1993 2002 [PM:9560412] 9560412 10.1093/genetics/148.4.1993 PMC1460107

[B17] Parsch J Braverman J Stephan W Comparative sequence analysis and patterns of covariation in RNA secondary structures Genetics 2000 154 2 909 921 [ISI:000085178700036] 10655240 10.1093/genetics/154.2.909 PMC1460946

[B18] Jow H Hudelot C Rattray M Higgs P Bayesian phylogenetics using an RNA substitution model applied to early mammalian evolution Mol Biol Evol 2002 19 9 1591 1601 [ISI:000178073700019] 12200486 10.1093/oxfordjournals.molbev.a004221

[B19] Telford M Wise M Gowri-Shankar V Consideration of RNA secondary structure significantly improves likelihood-based estimates of phylogeny: Examples from the bilateria Mol Biol Evol 2005 22 4 1129 1136 [ISI:000228139400033] 10.1093/molbev/msi099 15689526

[B20] Niehuis O Yen S Naumann C Misof B Higher phylogeny of zygaenid moths (Insecta : Lepidoptera) inferred from nuclear and mitochondrial sequence data and the evolution of larval cuticular cavities for chemical defence Mol Phylogenet Evol 2006 39 3 812 829 [ISI:000238155300016] 10.1016/j.ympev.2006.01.007 16483803

[B21] Dohrmann M Voigt O Erpenbeck D Worheide G Non-monophyly of most supraspecific taxa of calcareous sponges (Porifera, Calcarea) revealed by increased taxon sampling and partitioned Bayesian analysis of ribosomal DNA Mol Phylogenet Evol 2006 40 3 830 843 [PM:16762568] 10.1016/j.ympev.2006.04.016 16762568

[B22] Dohrmann M Janussen D Reitner J Collins A Worheide G Phylogeny and evolution of glass sponges (porifera, hexactinellida) Syst Biol 2008 57 3 388 405 [PM:18570034] 10.1080/10635150802161088 18570034

[B23] Erpenbeck D Nichols S Voigt O Dohrmann M Degnan B Hooper J Worheide G Phylogenetic analyses under secondary structure-specific substitution models outperform traditional approaches: case studies with diploblast LSU J Mol Evol 2007 64 5 543 557 [PM:17460808] 10.1007/s00239-006-0146-3 17460808

[B24] Fleck G Ullrich B Brenk M Wallnisch C Orland M Bleidissel S Misof B A phylogeny of anisopterous dragonflies (Insecta, Odonata) using mtRNA genes and mixed nucleotide/doublet models J Zool Syst Evol Res 2008 46 4 310 322 10.1111/j.1439-0469.2008.00474.x

[B25] Ware J May M Kjer K Phylogeny of the higher Libelluloidea (Anisoptera: Odonata): An exploration of the most speciose superfamily of dragonflies Mol Phylogenet Evol 2007 45 289 310 [PM:17728156] 10.1016/j.ympev.2007.05.027 17728156

[B26] von Reumont BM Meusemann K Szucsich NU Dell'Ampio E Gowri-Shankar V Bartel D Simon S Letsch HO Stocsits RR xia Luan Y Waegele JW Pass G Hadrys H Misof B Can comprehensive background knowledge be incorporated into substitution models to improve phylogenetic analyses? A case study on major arthropod relationships BMC Evol Biol 2009 9 119 10.1186/1471-2148-9-119 19473484 PMC2695459

[B27] Tsagkogeorga G Turon X Hopcroft RR Tilak MK Feldstein T Shenkar N Loya Y Huchon D Douzery EJP Delsuc F An updated 18S rRNA phylogeny of tunicates based on mixture and secondary structure models BMC Evol Biol 2009 9 187 10.1186/1471-2148-9-187 19656395 PMC2739199

[B28] Letsch HO Kuck P Stocsits RR Misof B The impact of rRNA secondary structure consideration in alignment and tree reconstruction: simulated data and a case study on the phylogeny of hexapods Mol Biol Evol 2010 msq140 http://mbe.oxfordjournals.org/cgi/content/abstract/msq140v1 10.1093/molbev/msq140 20530152

[B29] Keller A Förster F Müller T Dandekar T Schultz J Wolf M Including RNA secondary structures improves accuracy and robustness in reconstruction of phylogenetic trees Biol Direct 2010 5 4 10.1186/1745-6150-5-4 20078867 PMC2821295

[B30] Stocsits RR Letsch H Hertel J Misof B Stadler PF Accurate and efficient reconstruction of deep phylogenies from structured RNAs Nucleic Acids Res 2009 gkp600 http://nar.oxfordjournals.org/cgi/content/abstract/gkp600v1 10.1093/nar/gkp600 PMC2764418 19723687

[B31] Stamatakis A Ludwig T Meier H RAxML-III: a fast program for maximum likelihood-based inference of large phylogenetic trees Bioinformatics 2005 21 4 456 463 [PM:15608047] 10.1093/bioinformatics/bti191 15608047

[B32] Stamatakis A RAxML-VI-HPC: maximum likelihood-based phylogenetic analyses with thousands of taxa and mixed models Bioinformatics 2006 22 21 2688 2690 [PM:16928733] 10.1093/bioinformatics/btl446 16928733

[B33] Ott M Zola J Aluru S Stamatakis A Large-scale Maximum Likelihood-based Phylogenetic Analysis on the IBM BlueGene/L ACM/IEEE Supercomputing conference 2007 2007

[B34] Janies D Phylogenetic relationship of extant Echinoderm classes Can J Zool 2001 79 1232 1250 10.1139/z00-215

[B35] Littlewood D Smith A Clough K Emson R The interrelationships of the echinoderm classes: morphological and molecular evidence Biological Journal of the Linnean Society 1997 61 409 438 10.1111/j.1095-8312.1997.tb01799.x

[B36] Smith A Echinoderm larvae and phylogeny Annual Review of Ecology and Systematics 1997 28 219 241 10.1146/annurev.ecolsys.28.1.219

[B37] Smith A Paul C, Smith A Fossil evidence for the relationship of extant echinoderm classes and their times of divergence Echinoderm Phylogeny and Evolutionary Biology 1988 Oxford: Clarendon Press 85 97

[B38] Scouras A Smith M The complete mitochondrial genomes of the sea lily Gymnocrinus richeri and the feather star Phanogenia gracilis: signature nucleotide bias and unique nad4L gene rearrangement within crinoids Mol Phylogenet Evol 2006 39 2 323 334 [PM:16359875] 10.1016/j.ympev.2005.11.004 16359875

[B39] Wada H Evolutionary history of free-swimming and sessile lifestyles in urochordates as deduced from 18S rDNA molecular phylogeny Mol Biol Evol 1998 15 9 1189 1194 9729883 10.1093/oxfordjournals.molbev.a026026

[B40] Swalla BJ Cameron CB Corley LS Garey JR Urochordates are monophyletic within the deuterostomes Syst Biol 2000 49 52 64 10.1080/10635150050207384 12116483

[B41] Kurabayashi A Okuyama M Ogawa M Takeuchi A Jing Z Naganuma T Saito Y Phylogenetic position of a deep-sea ascidian, Megalodicopia hians, inferred from the molecular data Zoolog Sci 2003 20 10 1243 1247 10.2108/zsj.20.1243 14569147

[B42] Tsagkogeorga G Turon X Galtier N Douzery EJP Delsuc F Accelerated evolutionary rate of housekeeping genes in tunicates J Mol Evol 2010 71 2 153 167 10.1007/s00239-010-9372-9 20697701

[B43] Zeng L Swalla B Molecular phylogeny of the protochordates: chordate evolution Can J Zool 2005 83 1 24 33 10.1139/z05-010

[B44] Zeng L Jacobs MW Swalla BJ Coloniality has evolved once in Stolidobranch Ascidians Integrative and Comparative Biology 2006 46 3 255 268 http://icb.oxfordjournals.org/content/46/3/255.abstract 10.1093/icb/icj035 21672740

[B45] Kjer K Honeycutt R Site specific rates of mitochondrial genomes and the phylogeny of eutheria BMC Evolutionary Biology 2007 7 8 16 [ISI:000244254600001] 10.1186/1471-2148-7-8 17254354 PMC1796853

[B46] Springer MS Cleven GC Madsen O de Jong WW Waddell VG Amrine HM Stanhope MJ Endemic African mammals shake the phylogenetic tree Nature 1997 388 6637 61 64 10.1038/40386 9214502

[B47] Stanhope MJ Madsen O Waddell VG Cleven GC de Jong WW Springer MS Highly congruent molecular support for a diverse superordinal clade of endemic African mammals Mol Phylogenet Evol 1998 9 3 501 508 10.1006/mpev.1998.0517 9667998

[B48] Madsen O Scally M Douady CJ Kao DJ DeBry RW Adkins R Amrine HM Stanhope MJ de Jong WW Springer MS Parallel adaptive radiations in two major clades of placental mammals Nature 2001 409 6820 610 614 10.1038/35054544 11214318

[B49] Murphy W Eizirik E Johnson W Zhang Y Ryder O O'Brien S Molecular phylogenetics and the origins of placental mammals Nature 2001 409 6820 614 618 [PM:11214319] 10.1038/35054550 11214319

[B50] Murphy W Eizirik E O'Brien S Madsen O Scally M Douady C Teeling E Ryder O Stanhope M de Jong W Springer M Resolution of the early placental mammal radiation using Bayesian phylogenetics Science 2001 294 5550 2348 2351 [PM:11743200] 10.1126/science.1067179 11743200

[B51] Hudelot C Gowri-Shankar V Jow H Rattray M Higgs P RNA-based phylogenetic methods: application to mammalian mitochondrial RNA sequences Mol Phylogenet Evol 2003 28 2 241 252 [ISI:000184530100006] 10.1016/S1055-7903(03)00061-7 12878461

[B52] Springer MS Teeling EC Madsen O Stanhope MJ de Jong WW Integrated fossil and molecular data reconstruct bat echolocation Proc Natl Acad Sci USA 2001 98 11 6241 6246 10.1073/pnas.111551998 11353869 PMC33452

[B53] Sullivan J Swofford D Are Guinea Pigs Rodents? The Importance of Adequate Models in Molecular Phylogenetics Journal of Mammalian Evolution 1997 4 2 77 86 10.1023/A:1027314112438

[B54] Springer MS DeBry RW Douady C Amrine HM Madsen O de Jong WW Stanhope MJ Mitochondrial versus nuclear gene sequences in deep-level mammalian phylogeny reconstruction Mol Biol Evol 2001 18 2 132 143 11158372 10.1093/oxfordjournals.molbev.a003787

[B55] Gibson A Gowri-Shankar V Higgs P Rattray M A comprehensive analysis of mammalian mitochondrial genome base composition and improved phylogenetic methods Mol Biol Evol 2005 22 2 251 264 [ISI:000226465100008] 15483324 10.1093/molbev/msi012

[B56] Xia X Xie Z DAMBE: software package for data analysis in molecular biology and evolution J Hered 2001 92 4 371 373 10.1093/jhered/92.4.371 11535656

[B57] Xia X Xie Z Kjer K 18S ribosomal RNA and tetrapod phylogeny Syst Biol 2003 52 3 283 295 [ISI:000182999600001] 10.1080/10635150390196948 12775520

[B58] Robinson DF Foulds LR Comparison of phylogenetic trees Math Biosci 1981 53 1-2 131 147 http://www.sciencedirect.com/science/article/B6VHX-45F633S-10/2/4f48e7845ed373b5259ac20b666f6364 10.1016/0025-5564(81)90043-2

[B59] Van de Peer Y Neefs JM De Rijk P De Wachter R Reconstructing evolution from eukaryotic small-ribosomal-subunit RNA sequences: Calibration of the molecular clock J Mol Evol 1993 37 2 221 232 10.1007/BF02407359 8411212

[B60] Philippe H Forterre P The rooting of the universal tree of life is not reliable J Mol Evol 1999 49 4 509 523 10.1007/PL00006573 10486008

[B61] Lopez P Forterre P Philippe H The Root of the Tree of Life in the Light of the Covarion Model Journal of Molecular Evolution 1999 49 4 496 508 10.1007/PL00006572 10486007

[B62] Swofford D Thorne J Felsenstein J Wiegmann B The topology-dependent permutation test for monophyly does not test for monophyly Syst Biol 1996 45 4 575 579 [ISI:A1996WK35000011] 10.1093/sysbio/45.4.575

[B63] Kjer K Blahnik R Holzenthal R Phylogeny of Trichoptera (caddisflies): Characterization of signal and noise within multiple datasets Syst Biol 2001 50 6 781 816 [ISI:000173246500003] 10.1080/106351501753462812 12116634

[B64] Hillis DM Inferring complex phylogenies Nature 1996 383 6596 130 131 10.1038/383130a0 8774876

[B65] Hillis DM Taxonomic sampling, phylogenetic accuracy, and investigator bias Syst Biol 1998 47 3 8 10.1080/106351598260987 12064238

[B66] Yang H Golenberg E Shoshani J Proboscidean DNA from museum and fossil specimens: an assessment of ancient DNA extraction and amplification techniques Biochem Genet 1997 35 5-6 165 179 [PM:9332711] 9332711 10.1023/a:1021902125382

[B67] Graybeal A Is it better to add taxa or characters to a difficult phylogenetic problem? Syst Biol 1998 47 9 17 [PM:12064243] 10.1080/106351598260996 12064243

[B68] Pollock D Zwickl D McGuire J Hillis D Increased taxon sampling is advantageous for phylogenetic inference Syst Biol 2002 51 4 664 671 [PM:12228008] 10.1080/10635150290102357 12228008 PMC2943957

[B69] Zwickl D Hillis D Increased taxon sampling greatly reduces phylogenetic error Syst Biol 2002 51 4 588 598 [PM:12228001] 10.1080/10635150290102339 12228001

[B70] Gillespie J Structure-Based Methods for the Phylogenetic Analysis of Ribosomal RNA Molecules 2005 http://repository.tamu.edu/bitstream/handle/1969.1/2580/etd-tamu-2005B-ENTO-Gillesp.pdf

[B71] Salemi M The phylogenetic handbook: a practical approach to DNA and protein phylogeny 2003 Cambridge University Press

[B72] Simon C Hewitt G, Johnston A, JPW Y Molecular systematics at the species boundary: exploiting conserved and variable regions of the mitochondrial genome of animals via direct sequencing from enzymatically amplified DNA In Molecular Techniques in Taxonomy 1991 New York: Springer Verlag, NATO Advanced Studies Institute 33 71

[B73] Mears J Sharma M Gutell R McCook A Richardson P Caulfield T Agrawal R Harvey S A structural model for the large subunit of the mammalian mitochondrial ribosome J Mol Biol 2006 358 193 212 [PM:16510155] 10.1016/j.jmb.2006.01.094 16510155 PMC3495566

[B74] Cannone J Subramanian S Schnare M Collett J D'Souza L Du Y Feng B Lin N Madabusi L Muller K Pande N Shang Z Yu N Gutell R The Comparative RNA Web (CRW) Site: an online database of comparative sequence and structure information for ribosomal, intron, and other RNAs: Correction (vol 3, pg 2, 2002) BMC Bioinformatics 2002 3 [ISI:000181476800015] 10.1186/1471-2105-3-2 PMC65690 11869452

[B75] Yusupov M Yusupova G Baucom A Lieberman K Earnest T Cate J Noller H Crystal structure of the ribosome at 5.5 angstrom resolution Science 2001 292 5518 883 896 [ISI:000168514900033] 10.1126/science.1060089 11283358

[B76] Gowri-Shankar V Rattray M A reversible jump method for Bayesian phylogenetic inference with a nonhomogeneous substitution model Mol Biol Evol 2007 24 6 1286 1299 [ISI:000247207700002] 10.1093/molbev/msm046 17347157

[B77] De Rijk P Wuyts J Van de Peer Y Winkelmans T De Wachter R The European Large Subunit Ribosomal RNA database Nucleic Acids Res 2000 28 177 178 [ISI:000084896300052] 10.1093/nar/28.1.177 10592218 PMC102430

[B78] Van de Peer Y De Rijk P Wuyts J Winkelmans T De Wachter R The European Small Subunit Ribosomal RNA database Nucleic Acids Res 2000 28 175 176 [ISI:000084896300051] 10.1093/nar/28.1.175 10592217 PMC102429

[B79] Wuyts J Van de Peer Y Wachter R Distribution of substitution rates and location of insertion sites in the tertiary structure of ribosomal RNA Nucleic Acids Res 2001 29 24 5017 5028 [ISI:000172871800015] 10.1093/nar/29.24.5017 11812832 PMC97625

[B80] Gowri-Shankar V Jow H PHASE: a software package for Phylogenetics And Sequence Evolution 2.0 2006 University of Manchester http://intranet.cs.man.ac.uk/ai/Software/phase/phase-2.0-manual.pdf

[B81] Katoh K Misawa K Kuma K Miyata T MAFFT: a novel method for rapid multiple sequence alignment based on fast Fourier transform Nucleic Acids Res 2002 30 14 3059 3066 [PM:12136088] 10.1093/nar/gkf436 12136088 PMC135756

[B82] Misof B Misof K A Monte Carlo Approach Successfully Identifies Randomness in Multiple Sequence Alignments : A More Objective Means of Data Exclusion Syst Biol 2009 58 21 34 http://sysbio.oxfordjournals.org/cgi/content/abstract/58/1/21 10.1093/sysbio/syp006 20525566

