# Supplementary material for: Potential pitfalls of modelling ribosomal RNA data in phylogenetic tree reconstruction: Evidence from case studies in the Metazoa
Source: BMC Evol Biol. 2011 May 27;11:146. doi: 10.1186/1471-2148-11-146 (PMC3123606; doi:10.1186/1471-2148-11-146)
Supplement: Additional file 1 — Additional tree reconstruction results. Detailed discussion on the results of tree reconstruction of Chilopoda, Hexapoda, Anisoptera, Primates and Heterobranchia. [file 1471-2148-11-146-S1.PDF]

Potential pitfalls of modelling ribosomal RNA data in phylogenetic tree reconstruction: Evidence from case studies in the Metazoa.

Harald O Letsch, Karl M Kjer

Supplement S3 - Additional tree reconstruction results

Phylogeny of Chilopoda

In the Chilopoda, five different clades have been recognised in the past, whose relationships are mostly well founded on complex morphological character systems [see 1]. Higher level centipede phylogeny splits Chilopoda into two different clades: the order Scutigeromorpha and Pleurostigmophora, the latter consisting of the remaining four centipede orders Lithobiomorpha, Craterostigmomorpha, Scolopendromorpha and Geophilomorpha. The two latter ones confirm a monophyletic clade, Epimorpha, defined by a complete segmentation during embryogenesis. Due to maternal brood care, Craterostigmomorpha have been proposed as sister group to Epimorpha [see 2]. If assessed by either of these outlined hypotheses on chilopod phylogeny, only two tree reconstruction methods were able to provide a reliable result: The GTR and the RNA6A setups show a basal split between Scutigeromorpha and Pleurostigmophora, monophyletic Epimorpha (Scolopendromorpha and Geophilomorpha) and Craterostigmomorpha as sister group to all other Pleurostigmophora. In contrast, all other mixed RNA/DNA setups show monophyletic (RNA6B-D, RNA7A-F) or even paraphyletic (all RNA16 model setups) Geophilomorpha as first(s) split within Chilopoda (Fig. 1). According to previous hypotheses of chilopod evolution, these hypotheses are most probably an artifact.

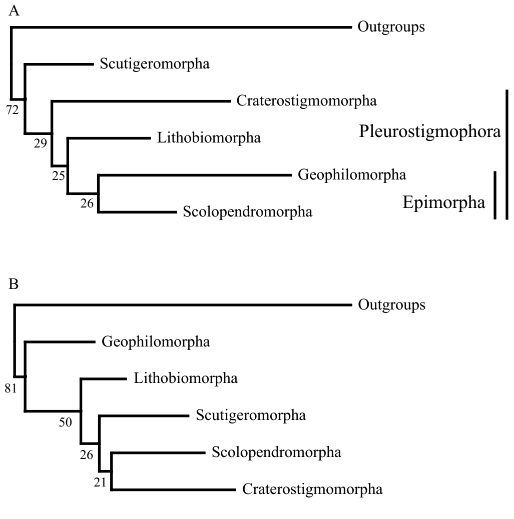

Figure 1: Trees summarising the results of the tree reconstructions on Chilopoda. (A) Tree by GTR and RNA6A model setups with monophyletic Pleurostigmophora and Epimorpha. Bootstrap (BS) values taken from the GTR model based tree reconstruction. (B) Tree and Bootstrap (BS) values derived from the RNA6B model setup as an example of most mixed RNA/DNA model setups, showing basal (paraphyletic) Geophilomorpha and Scutigeromorpha nested within Pleurostigmophora.

Phylogeny of hexapod orders

The relationships of hexapod orders are clearly one of the most difficult challenges in arthropod systematics. The incongruencies of many morphological and molecular analyses concerning hexapod relationships [e.g. 3–9] have been explained by the "ancient rapid radiation" phenomenon [10–12], which describes the appearance of most hexapod lineages in a geologically relatively short time span in the upper Devonian,

followed by a long period, in which these lineages have evolved separately, further complicated by the fact that most extant insects are much younger than their stem lineages. Nevertheless, there is consensus on several relationships of the backbone of hexapod phylogeny, e.g. the basal split into Entognaths and Ectognaths and the monophyly of Dicondylia (Zygentoma + Pterygota), which allows assessment of tree reconstruction results. According to that, all results of the present hexapod analyses must be seen as incorrect (Fig. 2). This is due to the probably incorrect position of the zygentome species *Lepisma saccharina*, which either appears within Entognatha (RNA7A-F, RNA16,A,B) or as sistergroup to Neoptera (GTR, RNA6A-D). As the support for *Lepisma saccharina* + Neoptera is very low, the basal splits within Dicondylia are virtually a trichotomy. Thus, the DNA and RNA6 model setups might be seen as slightly superior to the RNA7 and RNA16 models. However, this data set is taken from [9] and it is notable, that in the Bayesian Inference analyses applied there, the DNA model setup led to the expected phylogeny (Archeognatha(Zygentoma(”Palaeoptera”(Neoptera)))).

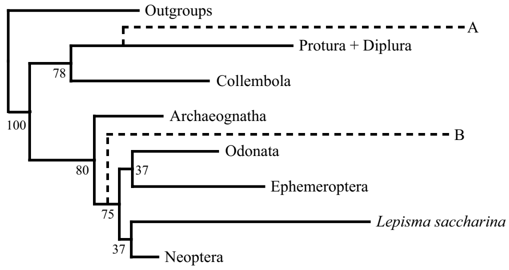

Figure 2: Tree summarising the results of all tree reconstructions on Hexapoda. The dotted line (A) indicates the alternative position of the *Lepisma saccharina*, whereas (B) indicates its expected position. BS values taken form the GTR model based analyses.

Phylogeny of anisopteran families

Within Anisoptera, species are classified into 13 families, clearly forming monophyletic groups, well characterised by morphological autapomorphies. Among these groups, the relationships remain disputed. Traditional phylogenetic studies, [13–20], are mostly hampered by homoplastic data sets, lack of a sufficient outgroup or non-cladistic ”analyses”. Recent morphological and molecular studies on anisopteran inter-family relationships [21–24] propose monophyly of the superfamily Libelluloidea comprising all taxa with larval spoon-shaped mask and a basal position of Aeschnomorpha (Aeshnidae + Austropetaliidae). In contrast, it remains unclear if either Gomphidae or Petaluridae are the closest relative to Libelluloidea. This situation is reflected by the results of the current analyses of nc and nt rRNA data of Anisoptera, displaying the following relationships: (Aeshnomorpha(Gomphidae,Petaluridae(Libelluloidea))). Nevertheless, the DNA model setup, as well as several mixed RNA/DNA setups (RNA6B-D, RNA7D and RNA16B) show a dubious sistergroup relationship between Gomphidae and Petaluridae, which has never been supported in any previous study on anisopteran phylogeny and might be an artifact of tree reconstruction (Fig. 3).

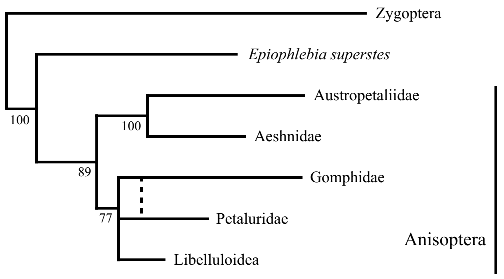

Figure 3: Tree summarising the results of all tree reconstructions on Anisoptera. Dotted line indicates alternative sistergroup relationship of Gomphidae and Petaluridae, displayed by the following model setups: GTR, RNA6B-D, RNA7D and RNA16B. BS values taken form the GTR model based analyses.

The position of *Tarsius* within Primates

We focus on the phylogeny of primates and, in particular, on the exact phylogenetic position of one of the most basal primates, the tarsier. Molecular studies on mitochondrial and nuclear DNA data of primates have so far lead to incongruent results. Nuclear DNA data favour haplorrhines (“dry-nosed” primates), i.e. the grouping of anthropoids (human-like apes) and tarsier [25]. Mitochondrial data,

in contrast, mostly support the "prosimian hypothesis" that postulates a sister group relationships of *Tarsius* and strepsirrhines ("wet-nosed" primates) [26–28]. However, the latter hypothesis has been strongly contradicted by the discovery of haplorrhine-specific SINES [29, 30]. In our present analyses, nearly all trees are congruent to the "haplorrhine hypothesis", with the exception of the RNA6D model setup, displaying the "prosimian hypothesis" (Fig. 4). Similar to the mammalian data set, the support values for deep nodes are increasing, if mixed RNA/DNA models are applied.

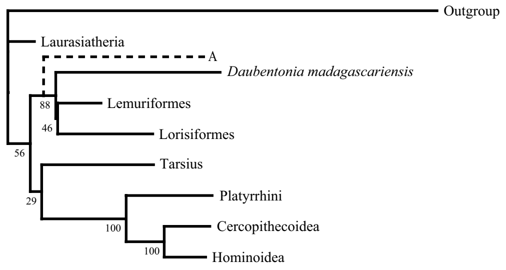

Figure 4: Tree summarising the results of all tree reconstructions on Primates, except the RNA6D model. Dotted line (A) indicates alternative sistergroup relationship of *Tarsius* and Strepsirrhini, displayed by the RNA6D model setup. BS values taken form the GTR model based analyses.

Relationships within Heterobranchia

Phylogenetic relationships of Heterobranchia, which represents the most diverse and speciose group within Gastropoda, are recently only poorly resolved. Morphological data provide robust signal only for the monophyly of Pulmonata, whereas its support for Euthyneura (Opisthobranchia + Pulmonata) and Opistobranchia is only weak [31–34]. This is reflected by recent molecular studies on the phylogeny of heterobranch snails [e.g. 35–41]. For our present work, we adopted the data set of [41], who applied 28S sequence data on the phylogeny of Pulmonata. The different model setups are congruent regarding the monophyly of Euthyneura and Eupulmonata. Nevertheless, there is a remarkable difference between the position of *Aplysia californica*. In most mixed RNA/DNA model setups (RNA6A-D,RNA7A,RNA16-A), this opisthobranch species clusters together with *Boonea seminuda* within Pulmonata (Fig. 5). Although the remaining mixed RNA/DNA model setups provide a more reasonable tree hypothesis with the opistobranch species paraphyletic to Pulmonata at the base of Euthyneura, the monophyly of Opistobranchia within in the GTR model setup is the only tree hypothesis compatible with recent molecular studies on the phylogeny of Opisthobranchia. Although polyphyly of Opisthobranchia is mainly accepted, mostly due to the position of Sacoglossa, the opisthobranch species included here, are accepted as a monophyletic subgroup within Opisthobranchia [37, 39, 42, 43].

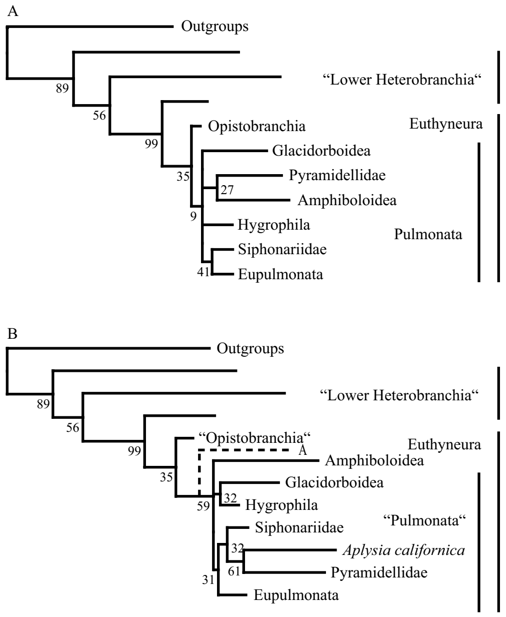

Figure 5: Trees summarising the results of the tree reconstructions on Heterobranchia. (A) Tree and Bootstrap (BS) values of GTR model setups with monophyletic Opisthobranchia and Pulmonata. (B) Tree derived from the mixed RNA/DNA model setups, showing paraphyletic Opisthobranchia and *Aplysia californica* nested within Pulmonata. The dotted line (A) indicates an alternative position of *Aplysia californica*. BS values taken form the RNA16 model based analyses.

References

[1] Murienne J, Edgecombe GD, Giribet G. Including secondary structure, fossils and molecular dating in the centipede tree of life. Mol.Phylogenet.Evol. 2010;57(1):301–313. doi:10.1016/j.ympev.2010.06.022.

[2] Edgecombe GD, Giribet G. Evolutionary biology of centipedes (Myriapoda: Chilopoda). Annu Rev Entomol. 2007;52:151–170. doi:10.1146/annurev.ento.52.110405.091326.

[3] Kjer KM. Aligned 18S and insect phylogeny. Syst.Biol. 2004;53(3):506–514. doi:10.1080/10635150490445922.

[4] Yoshizawa K, Johnson KP. Aligned 18S for Zoraptera (Insecta): phylogenetic position and molecular evolution. Mol.Phylogenet.Evol. 2005;37(2):572–580. doi:10.1016/j.ympev.2005.05.008.

[5] Beutel RG, Gorb SN. Ultrastructure of attachment specializations of hexapods (Arthropoda): evolutionary patterns inferred from a revised ordinal phylogeny. J.Zool.Syst.Evol.Res. 2001;39(4):177–207. doi:10.1046/j.1439-0469.2001.00155.x.

[6] Beutel RG, Pohl H. Endopterygote systematics - Where do we stand and what is the goal (Hexapoda, Arthropoda)? Syst.Entomol. 2006;31(2):202–219. doi:10.1111/j.1365-3113.2006.00341.x.

[7] von Reumont BM, Meusemann K, Szucsich NU, Dell'Ampio E, Gowri-Shankar V, Bartel D, et al. Can comprehensive background knowledge be incorporated into substitution models to improve phylogenetic analyses? A case study on major arthropod relationships. BMC Evol.Biol. 2009;9:119. doi:10.1186/1471-2148-9-119.

[8] Meusemann K, von Reumont BM, Simon S, Roeding F, Strauss S, Kuck P, et al. A phylogenomic approach to resolve the arthropod tree of life. Mol.Biol.Evol. 2010;27(11): 2451–2464 . doi:10.1093/molbev/msq130.

[9] Letsch HO, Kuck P, Stocsits RR, Misof B. The impact of rRNA secondary structure consideration in alignment and tree reconstruction: simulated data and a case study on the phylogeny of hexapods. Mol.Biol.Evol. 2010;27(11):2507–2521. doi:10.1093/molbev/msq140.

[10] Whitfield JB, Lockhart PJ. Deciphering ancient rapid radiations. Trends Ecol.Evol. 2007;22(5):258–265. doi:10.1016/j.tree.2007.01.012.

[11] Whitfield JB, Kjer KM. Ancient rapid radiations of insects: challenges for phylogenetic analysis. Annu.Rev.Entomol. 2008;53:449–472. doi:10.1146/annurev.ento.53.103106.093304.

[12] Rokas A, Carroll SB. Bushes in the tree of life. PLoS Biol. 2006;4(11):e352. doi:10.1371/journal.pbio.0040352.

[13] Fraser FC. A reclassification of the order Odonata. Handbook of the Royal Zoological Society of New South Wales. 1957;12:1–134.

[14] Pfau HK. Contributions of functional morphology to the phylogenetic systematics of Odonata. Adv.Odonatol. 1991;5:109–141.

[15] Carle FL. Evolution, taxonomy, and biogeography of ancient Gondwanian libelluloides, with comments on anisopteroid evolution and phylogenetic systematics (Anisoptera: Libelluloidea). Odonatologica. 1995;24(4):383–506.

[16] Bechly G. Morphologische Untersuchungen am Flügelgeäder der rezenten Libellen und deren Stammgruppenvertreter (Insecta: Odonatoptera), unter besonderer Bercksichtigung der Phylogenetischen Systematik und des Grundplanes der Odonata. Petalura. 1996;2:1–402.

[17] Trueman J. A preliminary cladistic analysis of odonate wing venation. Odonatologica. 1996;25(1):59–72.

[18] Lohmann H. Das Phylogenetische System der Anisoptera (Odonata). Entomol. Zeitschrift. 1996;106(6): 209-252; 106(7): 253-266; 106(9): 360-367.

[19] Bechly G. Phylogenetic systematics of Odonata. Website on Internet; 2002. http://www.bernstein.naturkundemuseum-bw.de/odonata/phylosys.htm.

[20] Pfau HK. Structure, function and evolution of the glans of the anisopteran vesica spermalis (Odonata). Odonatologica. 2005;8(2):259–310.

[21] Carle FL, Kjer KM. Phylogeny of *Libellula* Linnaeus (Odonata: Insecta). Zootaxa. 2002;85:1–18.

[22] Carle F, Kjer KM, May ML. Evolution of Odonata, with Special Reference to Coenagrionoidea (Zygoptera). Arthropod Syst.Phylogeny. 2008;66(1):37–44.

[23] Bybee SM, Ogden TH, Branham MA, Whiting MF. Molecules, morphology and fossils: a comprehensive approach to odonate phylogeny and the evolution of the odonate wing. Cladistics. 2008;24(4):477–514. doi:10.1111/j.1096-0031.2007.00191.x.

[24] Letsch HO, Schmidt C, Kück P, Fleck G, Stocsists RR, Misof B. Simultaneous alignment and folding of 28S rRNA sequences uncovers phylogenetic signal in structure variation. Mol.Phylogenet.Evol. 2009;53(3):758–771. doi:10.1016/j.ympev.2009.07.033.

[25] Goodman M, Porter CA, Czelusniak J, Page SL, Schneider H, Shoshani J, et al. Toward a phylogenetic classification of Primates based on DNA evidence complemented by fossil evidence. Mol.Phylogenet.Evol. 1998;9(3):585–598. doi:10.1006/mpev.1998.0495.

[26] Hayasaka K, Gojobori T, Horai S. Molecular phylogeny and evolution of primate mitochondrial DNA. Mol.Biol.Evol. 1988;5(6):626–644.

[27] Murphy WJ, Eizirik E, Johnson WE, Zhang YP, Ryder OA, O'Brien SJ. Molecular phylogenetics and the origins of placental mammals. Nature. 2001;409(6820):614–618. doi:10.1038/35054550.

[28] Schmitz J, Ohme M, Zischler H. The complete mitochondrial sequence of *Tarsius bancanus*: evidence for an extensive nucleotide compositional plasticity of primate mitochondrial DNA. Mol.Biol.Evol. 2002;19(4):544–553.

[29] Zietkiewicz E, Richer C, Labuda D. Phylogenetic affinities of tarsier in the context of primate Alu repeats. Mol.Phylogenet.Evol. 1999;11(1):77–83. doi:10.1006/mpev.1998.0564.

[30] Schmitz J, Ohme M, Zischler H. SINE insertions in cladistic analyses and the phylogenetic affiliations of *Tarsius bancanus* to other primates. Genetics. 2001;157(2):777–784.

[31] Haszprunar G. The Heterobranchia a new concept of the phylogeny of the higher Gastropoda. Z.Zool.Syst.Evolutionsforsch. 1985;23:15–37.

[32] Haszprunar G. On the origin and evolution of major gastropod groups, with special reference to the Streptoneura. J.Moll.Studies. 1988;54(4):367–441. doi:10.1093/mollus/54.4.367.

[33] Ponder WF, Lindberg DR. Towards a phylogeny of gastropod molluscs: an analysis using morphological characters. Zool.J.Linn.Soc. 1997;119(2):83–265.

[34] Dayra B, Tillier S. Evolutionary relationships of euthyneuran gastropods (Mollusca): a cladistic re-evaluation of morphological characters. Zool.J.Linn.Soc. 2002;135(4):403–470.

[35] Colgan DJ, Ponder WF, Beacham E, Macaranas JM. Gastropod phylogeny based on six segments from four genes representing coding or non-coding and mitochondrial or nuclear DNA. Moll.Res. 2003;23(2):123–148. doi:10.1071/MR03002.

[36] Dayrat B, Tillier A, Lecointre G, Tillier S. New clades of euthyneuran gastropods (Mollusca) from 28S rRNA sequences. Mol.Phylogenet.Evol. 2001;19(2):225–235. doi:10.1006/mpev.2001.0926.

[37] Grande C, Templado J, Cervera JL, Zardoya R. Molecular phylogeny of Euthyneura (Mollusca: Gastropoda). Mol.Biol.Evol. 2004;21(2):303–313. doi:10.1093/molbev/msh016.

[38] Grande C, Templado J, Zardoya R. Evolution of gastropod mitochondrial genome arrangements. BMC Evol.Biol. 2008;8:61. doi:10.1186/1471-2148-8-61.

[39] Klussmann-Kolb A, Dinapoli A, Kuhn K, Streit B, Albrecht C. From sea to land and beyond–new insights into the evolution of euthyneuran Gastropoda (Mollusca). BMC Evol.Biol. 2008;8:57. doi:10.1186/1471-2148-8-57.

[40] Dinapoli A, Klussmann-Kolb A. The long way to diversity–phylogeny and evolution of the Heterobranchia (Mollusca: Gastropoda). Mol.Phylogenet.Evol. 2010;55(1):60–76. doi:10.1016/j.ympev.2009.09.019.

[41] Holznagel WE, Colgan DJ, Lydeard C. Pulmonate phylogeny based on 28S rRNA gene sequences: a framework for discussing habitat transitions and character transformation. Mol.Phylogenet.Evol. 2010;57(3):1017–1025. doi:10.1016/j.ympev.2010.09.021.

[42] Vonnemann V, Schroedl M, Klussmann-Kolb A, Waegele H. Reconstruction of the phylogeny of the Opisthobranchia (Mollusca: Gastropoda) by means of 18s and 28s rRNA gene sequences. J.Moll.Studies. 2005;71(2):113–125.

[43] Waegele H, Klussmann-Kolb A. Opisthobranchia (Mollusca, Gastropoda) - more than just slimy slugs. Shell reduction and its implications on defence and foraging. Front Zool. 2005;2(1):3. doi:10.1186/1742-9994-2-3.
