# Supplementary material for: Potential pitfalls of modelling ribosomal RNA data in phylogenetic tree reconstruction: Evidence from case studies in the Metazoa
Source: BMC Evol Biol. 2011 May 27;11:146. doi: 10.1186/1471-2148-11-146 (PMC3123606; doi:10.1186/1471-2148-11-146)
Supplement: Additional file 3 — Tables. Tables providing the Genbank accession numbers of constraint sequences used for the RNASALSA alignment, the detailed results of the AICc test and the detailed results of the Robinson-Foulds distance measurements. [file 1471-2148-11-146-S3.PDF]

Potential pitfalls of modelling ribosomal RNA data in phylogenetic tree reconstruction: Evidence from case studies in the Metazoa.

Harald O Letsch, Karl M Kjer

Additional file 3 - Tables

Table S1: Genbank accession numbers of constraint sequences used for the RNAsalsa alignment.

Taxon	Constraint	Genbank Acc.			
		12S	16S	18S	28S
Echinodermata	*Thalia democratica*	-	-	D14366	-
Tunicata	*Thalia democratica*	-	-	D14366	-
Chilopoda	*Okanagana utahensis*	-	-	U06478	-
Anisoptera	*Anopheles sp.*	L20934	L20934	-	L78065
Mammalia	*Bos taurus*	V00654	V00654	-	-
Primates	*Bos taurus*	V00654	V00654	-	-
Heterobranchia	*Thalia democratica*	-	-	-	AF158725

Table S2: Results of the AICc tests I.

		Chilopoda		Hexapoda		Echinodermata		Heterobranchia	
Model	K	-ln	AICc	-ln	AICc	-ln	AICc	-ln	AICc
GTR		-9974,94	-	-60391,86	-	-13066,79	-	-11876,72	-
6A	21	-7921,76	15886,02	-43499,23	87040,70	-9861,99	19766,53	-9058,56	18159,52
6B	9	-7968,12	15954,33	-43730,60	87479,24	-9924,27	19866,65	-9083,76	18185,60
6C	6	-7969,18	15950,40	-43734,09	87480,20	-9923,57	19859,19	-9082,86	18177,75
6D	5	-8048,48	16106,99	-45006,00	90032,03	-10005,11	20020,26	-9303,80	18617,62
7A	28	-9031,19	18119,29	-51210,99	102478,34	-11849,29	23755,55	-10129,28	20315,24
7B	25	-9059,93	18170,58	-51211,86	102474,01	-11851,15	23753,07	-10132,46	20315,47
7C	17	-9134,38	18303,09	-52204,87	104443,88	-11865,45	23765,26	-10205,96	20446,18
7D	11	-9134,38	18290,90	-51616,30	103254,66	-12019,42	24061,00	-10255,52	20533,15
7E	9	-9182,64	18383,38	-52828,78	105675,60	-12057,31	24132,74	-10472,14	20962,36
7F	8	-9135,20	18286,47	-51615,58	103247,19	-12018,52	24053,12	-10254,95	20525,95
16	120	-9305,17	18867,41	-54548,43	109343,56	-12428,41	25115,14	-10317,13	20887,07
16A	21	-9534,81	19112,13	-55480,83	111003,90	-12760,77	25564,09	-10566,17	21174,72
16B	17	-10089,27	20212,87	-59711,77	119457,68	-13661,39	27357,14	-11068,61	22171,47

Table S3: Results of the AICc tests II.

		Tunicata		Primates		Mammalia		Anisoptera	
Model	K	-ln	AICc	-ln	AICc	-ln	AICc	-ln	AICc
GTR		-14532,94	-	-21871,75	-	-87274,27	-	-54156,28	-
6A	21	-11739,50	23521,48	-18262,48	36567,65	-67716,51	135475,31	-44326,97	88696,11
6B	9	-11791,21	23600,51	-18311,93	36641,99	-67887,39	135792,84	-44514,39	89046,81
6C	6	-11794,13	23600,30	-18311,56	36635,17	-67885,92	135783,87	-44513,85	89039,72
6D	5	-11976,77	23963,57	-18843,08	37696,20	-69281,41	138572,84	-44949,14	89908,30
7A	28	-13148,45	26351,71	-19798,87	39654,97	-75238,24	150532,98	-50050,59	100157,50
7B	25	-13153,34	26357,35	-19800,06	39651,08	-75249,05	150548,50	-50050,38	100151,02
7C	17	-13195,65	26425,61	-19965,12	39964,69	-75941,56	151917,32	-50212,42	100458,96
7D	11	-13321,19	26664,51	-20025,92	40074,03	-75952,04	151926,17	-50533,63	101089,30
7E	9	-13463,43	26944,95	-20192,44	40403,00	-76596,79	153211,63	-50779,45	101576,93
7F	8	-13322,29	26660,66	-20025,81	40067,72	-75958,08	151932,21	-50527,37	101070,77
16	120	-13558,69	27373,18	-20075,17	40413,74	-77912,13	156073,55	-51802,07	103849,81
16A	21	-13932,38	27907,23	-20572,55	41187,78	-79192,95	158428,18	-52856,37	105754,91
16B	17	-14576,25	29186,81	-21543,07	43120,59	-86353,20	172740,58	-56220,28	112474,67

Table S4: Results of the Robinson-Foulds distance measurements (unpaired positions).

	GTR	6A	6B	6C	6D	7A	7B	7C	7D	7E	7F	16	16A	16B
Chilopoda	30	34	44	44	38	50	50	46	48	50	48	52	56	54
Hexapoda	16	40	38	40	40	34	34	32	34	36	34	34	30	42
Echinodermata	154	132	138	128	142	156	154	152	158	162	162	148	158	168
Heterobranchia	32	14	14	14	14	20	22	20	24	20	24	30	18	10
Tunicata	52	32	32	32	28	36	34	36	38	42	38	32	36	52
Primates	10	4	6	4	4	8	8	8	8	8	8	10	6	10
Mammalia	54	44	44	44	38	40	40	42	42	44	44	46	48	58
Anisoptera	54	58	54	54	50	50	46	48	50	52	52	44	44	60

Table S5: Results of the Robinson-Foulds distance measurements (paired positions).

	GTR	6A	6B	6C	6D	7A	7B	7C	7D	7E	7F	16	16A	16B
Chilopoda	64	70	68	68	72	68	66	68	64	64	62	66	62	62
Hexapoda	52	46	48	46	46	60	60	56	60	58	60	60	62	60
Echinodermata	176	190	182	186	184	182	190	190	192	188	184	186	184	174
Heterobranchia	46	48	48	48	48	46	46	48	46	48	46	44	46	50
Tunicata	66	78	78	78	82	76	80	80	76	74	78	76	78	74
Primates	24	26	26	26	24	24	24	24	24	24	24	24	24	24
Mammalia	66	68	70	70	66	70	70	70	70	76	70	66	70	60
Anisoptera	76	80	78	78	74	70	72	76	74	72	70	76	74	66
