# Supplementary material for: Potential pitfalls of modelling ribosomal RNA data in phylogenetic tree reconstruction: Evidence from case studies in the Metazoa
Source: BMC Evol Biol. 2011 May 27;11:146. doi: 10.1186/1471-2148-11-146 (PMC3123606; doi:10.1186/1471-2148-11-146)
Supplement: Additional file 4 — Taxa list. A list of all applied sequence data with according Genbank accession numbers. [file 1471-2148-11-146-S4.PDF]

Potential pitfalls of modelling ribosomal RNA data in phylogenetic tree reconstruction: Evidence from case studies in the Metazoa.

Harald O Letsch, Karl M Kjer

Additional file 4 - Genbank accession numbers

Table S1: Species names and Genbank accession numbers of Echinodermata (18S).

Taxon	Species	Genbank Acc.
**Outgroups**		
Hemichordata	*Balanoglossus carnosus*	D14359
Tunicata	*Doliolum denticulata*	FM244861
	Thalia democratica	D14366
Vertebrata	*Anolis carolinensis*	AY859624
	Xenopus laevis	X04025
	Gallus gallus	AF173612
	Homo sapiens	X03205
**Echinodermata**		
Crinoidea	*Antedon serrata*	D14357
	Capillaster multiradiatus	AH008328
	Colobometra perspinosa	GQ913338
	Comanthina schlegelii	GQ913333
	Comanthus mirabilis	GQ913329
	Caledonicrinus vaubani	AY275892
	Dorometra aegyptica	AF088803
	Himerometra magnipinna	GQ913343
	Himerometra robustipinna	GQ913332
	Clarkcomanthus littoralis	GQ913344
	Endoxocrinus alternicirrus	GQ913341
	Guillecrinus neocaledonicus	AY275893
	Promachocrinus kerguelensis	GQ913342
	Phanogenia gracilis	GQ913331
	Oxycomanthus bennetti	GQ913330
	Liparometra articulata	GQ913335
	Metacrinus rotundus	GQ913339
	Neogymnocrinus richeri	AY275895
	Bathycrinus sp.	AY275891
	Holopus alidis	AY275896
Ophiuroidea	*Ophiocoma echinata*	DQ060802
	Ophioderma cenereum	AY859645
	Ophiomyxa flaccida	DQ060804
	Ophionereis reticulata	DQ060805
	Ophiopholis aculeata	DQ060806
	Ophioplocus japonicus	D14361
	Ophiopsammus maculata	DQ060807
	Ophiothrix oerstedii	DQ060808
	Asteronyx sp.	DQ060809
	Gorgonocephalus eucnemis	DQ060790
	undefined ophiuroid species	L28056*
	Schizostella bifurcata	DQ060818
Asteroidea	*Acanthaster planci*	AB084554
	Acodontaster conspicuus	DQ060774
	Allostichaster sp.	DQ060775
	Aphelasterias japonica	AB125600
	Archaster angulatus	AB084558
	Archaster typicus	AB084547
	Asterias forbesii	DQ077937
	Asterina batheri	AB084552
	Asterodon dilatatus	DQ060778
	Astrogonium miliarus	DQ060780
	Astrometis sertulifera	AY935545
	Astropecten polycanthus	DQ060782
	Coronaster marchenus	AY935546
	Coscinasterias acutispina	AB125601
	Crossaster papposus	AB084556
	Dermasterias imbricata	DQ060784
	Diplasterias brucei	DQ060785
	Echinaster sentus	DQ060786
	Evasterias troschelii	DQ060788
	Heliaster helianthoides	AF088804
	Hymenaster pellucidus	DQ060792
	Labidiaster annulatus	AY935548
	Leiaster leachi	AB084561

Table S1: Continued.

Taxon	Species	Genbank Acc.
	Leptasterias polaris	AY935556
	Luidia maculata	AB084548
	Macroptychaster accrescens	DQ060797
	Marthasterias glacialis	AY935547
	Mediaster aequalis	DQ060798
	Myxoderma platyacanthum	DQ060799
	Notasterias pedicellaris	AY935549
	Odontaster validus	DQ060801
	Orthasterias koehleri	DQ060810
	Lethasterias nanimensis	AY935563
	Perknaster fuscus	DQ060812
	Pisaster giganteus	AY935555
	Psalidaster mordax	AY935551
	Pseudarchaster parelli	AF088806
	Pteraster tesselatus	DQ060817
	Rathbunaster californicus	AF088807
	Solaster stimpsonii	DQ060819
	Stegnaster inflatus	DQ060820
	Stephanasterias albula	AY935564
	Stereocidaris excavatus	DQ073795
	Stichaster australis	DQ060821
	Stylasterias forreri	AY935562
	Tarsaster alaskanus	AY935568
	Urasterias lincki	AY935553
	Linckia laevigata	DQ060794
	Patiriella regularis	DQ060811
	Pedicellaster magister	AY935569
	Odinella nutrix	AY935550
	Pycnopodia helianthoides	AY935565
	Certonardoa semiregularis	AB084550
	Henricia ohshimai	AB084555
	Eurygonius hylacanthus	DQ060787
	Brisingaster robillardi	AF088802
Holothuroidea	*Actinopyga miliaris*	AY133476
	Afrocucumis africana	AY133483
	Bohadschia marmorata	AY133478
	Cucumaria elongata	AY133479
	Aslia lefevrei	AY133480
	Chiridota rotifera	DQ777081
	Apostichopus japonicus	D14364
	Holothuria edulis	AY133471
	Neopentadactyla mixta	AY133482
	Parastichopus californicus	DQ777084
	Pseudostichopus villosus	DQ777086
	Thyonella gemmata	AY133481
	Labidoplax digitata	AY133469
	Leptosynapta clarkii	DQ777083
	Pentacta pygmaea	DQ777085
Echinoidea	*Abatus cavernosus*	DQ073775
	Amblypneustes formosus	AF279212
	Amblypneustes ovum	AF279207
	Anthocidaris crassispina	DQ073776
	Araeosoma fenestratum	DQ073777
	Arbacia punctulata	DQ073778
	Archaeopneustes hystrix	DQ073779
	Aspidodiadema jacobi	DQ073780
	Caenopedina cubensis	DQ073781
	Calocidaris micans	DQ073782
	Centrostephanus longispinus	DQ073783
	Coelopleurus floridanus	DQ073784
	Conolampas sigsbei	DQ073785
	Cyrtechinus verruculatus	DQ073786
	Echinocyamus pusillus	DQ073787
	Echinolampas crassa	DQ073788
	Echinoneus cyclostomus	DQ073789
	Genocidaris maculata	AF279199
	Holopneustes porosissimus	AF279208
	Lytechinus variegatus	DQ073790
	Mespilia globulus	AF279203
	Microcyphus annulatus	AF279216
	Paleopneustes cristatus	DQ073791
	Plexechinus planus	AY957468
	Prionocidaris bispinosa	DQ073792
	Psammechinus miliaris	AF279215
	Pseudechinus novaezealandiae	AF279205
	Rumphia orbicularis	DQ073793
	Salmaciella oligopora	AF279211
	Salmacis belli	AF279213
	Spatangus raschi	DQ073794
	Stomopneustes variolaris	AF279214
	Temnopleurus alexandri	AF279206
	Temnotrema sculptum	AF279201

* Sequence with Acc. L28056 is erroneously addressed as *Strongylocentrotus purpuratus* in Genbank.

Table S2: Species names and Genbank accession numbers of Tunicata (18S).

Taxon	Species	Genbank Acc.
**Outgroups**		
Echinodermata	*Antedon serrata*	D14357
	Asterias amurensis	D14358
	Strongylocentrotus purpuratus	L28055
Hemichordata	*Balanoglossus carnosus*	D14359
	Ptychodera flava	AF278681
	Saccoglossus pusillus	AF236800
Cephalochordata	*Branchiostoma floridae*	M97571
Vertebrata	*Anolis carolinensis*	AY859624
	Chrysemys sp.	AY859627
	Danio rerio	BX296557
	Gallus gallus	AF173612
	Homo sapiens	X03205
	Petromyzon marinus	M97575
	Raja schmidti	AF278682
	Xenopus laevis	X04025
**Urochordata**		
Ascidiacea		
Aplousobranchia		
Clavelinidae	*Clavelina meridionalis*	FM244840
	Pycnoclavella aff. detorta	FM244841
Didemnidae	*Cystodytes sp.*	FM244842
	Didemnum molle	AB211071
	Didemnum sp.	AB211072
	Didemnum sp.	AB211073
	Diplosoma mitsukurii	AB211096
	Diplosoma ooru	AB211097
	Diplosoma simile	AB211101
	Diplosoma simileguwa	AB211107
	Diplosoma sp.	AB211116
	Diplosoma sp.	AB211121
	Diplosoma virens	AB211109
	Leptoclinides madara	AB211070
	Lissoclinum badium	AB211078
	Lissoclinum bistratum	AB211079
	Lissoclinum patella	AB211085
	Lissoclinum punctatum	AB211086
	Lissoclinum sp.	AB211095
	Lissoclinum timorense	AB211092
	Trididemnum paracyclops	AB211074
Polycitoridae	*Distaplia dubia*	AB211068
	Eudistoma gilboviride	AB211069
Polyclinidae	*Aplidium pliciferum*	AB211067
Phlebobranchia		
Ascidiidae	*Ascidia ahodori*	AB104871
	Ascidia ceratodes	L12378
	Ascidiella sp.	FM244843
	Phallusia fumigata	FM244844
	Phallusia mammillata	AF236803
	Phallusia nigra	FM244845
Cionidae	*Ciona intestinalis*	AB013017
	Ciona savignyi	AB191429
Corellidae	*Chelyosoma siboja*	AB104872
	Corella inflata	AY903930
	Corella eumyota	FM244846
Perophoridae	*Ecteinascidia herdmanni*	FM244847
	Ecteinascidia turbinata	FM244848
	Perophora sagamiensis	AB104873
	Perophora viridis	FM244849
Octacnemidae	*Megalodicopia hians*	AB075543
Stolidobranchia		
Molgulidae	*Molgula arenata*	AY903919
	Molgula bleizi	L12418
	Molgula citrina	L12420
	Molgula complanata	L12422
	Molgula manhattensis	L12426
	Molgula occidentalis	FM244850
	Molgula occulta	L12430
	Molgula oculata	L12432
	Molgula pacifica	AY040738
	Molgula provisionalis	L12434
	Molgula pugetiensis	AY903920
	Molgula retortiformis	AY903921
Pyuridae	*Boltenia villosa*	AY903924
	Halocynthia igaboja	AY903925
	Halocynthia roretzi	AB013016
	Halocynthia spinosa	FM244851
	Herdmania curvata	AF165827
	Herdmania momus #1	X53538
	Herdmania momus #2	AF165827
	Herdmania sp.	FM244852
	Microcosmus polymorphus	FM244853
	Microcosmus sabatieri	FM244854
	Microcosmus squamiger	FM244855
	Pyura dura	FM244856
	Pyura gangelion	FM244857

Table S2: Continued.

Taxon	Species	Genbank Acc.
	Pyura haustor	AY903926
Styelidae	*Botrylloides violaceus*	AY903927
	Botryllus planus	DQ346653
	Botryllus schlosseri #1	AB211066
	Botryllus schlosseri #2	FM244858
	Cnemidocarpa finmarkiensis	L12413
	Cnemidocarpa humilis	FM244859
	Dendrodoa grossularia	L12416
	Metandrocarpa taylori	AY903922
	Pelonaia corrugata	L12440
	Polycarpa mytiligera	FM244860
	Polycarpa papillata	DQ346654
	Polycarpa pomaria	L12441
	Styela gibbsii	AY903923
	Styela montereyensis	L12443
	Styela plicata	L12444
	Symplegma viride	DQ346655
Thaliacea		
Doliolida	*Doliolum denticulata*	FM244861
	Doliolum nationalis	AB013013
Pyrosomida	*Pyrosoma atlanticum*	AB013011
	Pyrosoma godeauxi	FM244862
	Pyrosomella verticillata	FM244863
Salpida	*Cyclosalpa quadriluminis*	FM244864
	Ihlea racovitzai	FM244865
	Salpa cylindrica	FM244866
	Salpa thompsoni	FM244867
	Thalia democratica	D14366
Appendicularia		
Oikopleuridae	*Megalocercus huxleyi*	FM244868
	Oikopleura dioica	AB013014
	Oikopleura labradoriensis	FM244869
	Oikopleura sp.	D14360

Table S3: Species names and Genbank accession numbers Chilopoda (18S).

Taxon	Species	Genbank Acc.
**Outgroups**		
Helminthomorpha	*Epicyliosoma sp.*	AF370785
	Narceus americanus	AY288686
	Polydesmus complanatus	EU368620
Penicillata	*Polyxenus lagurus*	EU368619
**Chilopoda**		
Scutigeromorpha	*Allothereua bidenticulata*	FJ660717
	Allothereua maculata	FJ660722
	Allothereua serrulata	DQ222130
	Dendrothereua homa	FJ660705
	Dendrothereua nubila	FJ660704
	Madagassophora hova	DQ222120
	Parascutigera festiva	FJ660725
	Parascutigera guttata	FJ660726
	Parascutigera latericia	FJ660731
	Parascutigera sphinx	FJ660739
	Pilbarascutigera incola	FJ660742
	Scutigera coleoptrata	FJ660712
	Scutigerina malagassa	DQ222119
	Scutigerina weberi	DQ222118
	Tachythereua sp.	FJ660716
	Thereuonema tuberculata	DQ222126
	Thereuonema turkestana	FJ660743
	Thereuopoda clunifera	AF119088
	Thereuopoda longicornis	DQ222125
	Thereuopodina n.	DQ222127
	Pselliodes guildingii	DQ222122
Craterostigmomorpha	*Craterostigmus crabilli*	EU024584
	Craterostigmus tasmanianus	EU368617
Lithobiomorpha	*Anopsobius giribeti*	AY509008
	Anopsobius neozelandicus	AY509005
	Anopsobius patagonicus	AY509007
	Bothropolys multidentatus	AF334272
	Dichelobius bicuspis	AY509010
	Dichelobius etnaensis	AY509009
	Dichelobius flavens	AY213720
	Dichelobius giribeti	AY213721
	Lamyctes africanus	AF334274
	Lamyctes brevilabiatus	AY213734
	Lamyctes coeculus	AY213735
	Lamyctes inermipes	AY213726
	Lamyctinus coeculus	AF334275
	Lithobius forficatus	EU368618
	Lithobius obscurus	AF334271
	Paralamyctes validus	AF334290

Table S3: Continued.

Taxon	Species	Genbank Acc.
	Paralamyctes weberi	AF334282
	Henicops maculatus	DQ201418
	Shikokuobius japonicus	AY213719
Scolopendromorpha	*Alipes crotalus*	AY288691
	Cryptops australis	AY288692
	Cryptops spinipes	AY288693
	Scolopendra multilans	AY336740
	Scolopendra viridis	DQ201419
	Scolopocryptops sexspinosus	AY288694
	Theatops posticus	AY288695
Geophilomorpha	*Bothriogaster signata*	AY288698
	Esastigmatobius japonicus	AF334291
	Eupolybothrus fasciatus	AY213718
	Geophilus electricus	AY288700
	Mecistocephalus guildingii	AY288696
	Pachymerium ferrugineum	AY288702
	Plesioschendyla confossa	AY288699
	Zelanophilus provocator	AY288701

Table S4: Species names and Genbank accession numbers to Hexapoda.

Taxon	Species	Genbank Acc. 18S	28S
**Outgroups**			
Tardigrada	*Milnesium tardigradum*	U49909	AY210826
Myriapoda	*Polyxenus lagurus*	EU368619	EU376011
Chelicerata	*Siro rubens*	AY428818	AY859602
	Limulus polyphemus	U91490	AF212167
Crustacea	*Squilla empusa*	L81946	AY210842
	Homarus americanus	AY743945	AY859581
**Hexapoda**			
Collembola	*Podura aquatica #1*	AY596363	AY210838
	Podura aquatica #2	EU368604	EF199970
	Allacma fusca	EU368610	EU376054
	Anurida maritima	EU368603	AJ251738
	Bilobella aurantiaca	EU368602	AJ251729
	Cryptopygus antarcticus	EU368605	EF199971
	Dicyrtomina saundersi	EU368611	EF199974
	Gomphiocephalus hodgsoni	EU368601	EF199969
	Isotoma viridis	AY596361	EU376052
	Megalothorax minimus	EU368608	EF199975
	Orchesella villosa	EU3686068	EF199972
	Triacanthella sp.	AY859610	AY859609
	Tetrodontophora bielanensis	AY555519	EU376051
	Pogonognathellus flavescens	EU368607	EU376053
	Sminthurus viridis #1	EU368609	EF199973
	Sminthurus viridis #2	AY859604	AY859603
Protura	*Acerentomon franzi*	EU368597	EF199976
	Baculentulus densus	Y037169	EU376049
	Eosentomon sakura	AY596355	EF192434
	Sinentomon erythranum	AY596358	EF192442
Diplura	*Campodeidae sp.*	AY859561	AY859560
	Catajapyx aquilonaris	EU368600	EF199978
	Campodea augens	EU368599	EF199977
	Lepidocampa weberi	AY037167	EU376050
	Octostigma sinensis	AY145134	EF192439
	Parajapyx emeryanus	AY037168	EF192440
Archaeognatha	*Lepismachilis y-signata*	EU368613	EF199980
	Machilis hrabei	EU368612	EF199981
	Pedetontus okaijmae	EU368614	EU376055
Zygentoma	*Lepisma saccharina*	EU368615	EU376048
Ephemeroptera	*Callibaetis ferrugineus*	AF370791	AY859557
	Epeorus sylvicola	AY749837	EU414715
	Siphlonura aestivalis	DQ008181	EU414716
Odonata	*Leucorrhinia sp.*	AY859584	AY859583
	Aeshna juncea	AF461231	EU424324
	Brachytron pratense	AF461232	EU424323
	Cordulia aenea	AF461236	EU424326
	Epiophlebia superstes	AF461247	EU424328
	Lestes viridis	AJ421949	EU424331
	Oxygastra curtisii	DQ008194	EU424325
	Progomphus obscurus	AY749909	EU424329
	Sympetrum danae	AF461243	EU424330
	Somatochlora flavomaculata	AF461242	EU424327
Demaptera	*Forficula auricularia*	Z97594	EU426876
Plecoptera	*Isoperla sp.*	AF461256	EU414717
	Nemoura flexuosa	AF461257	EU414718
Dictyoptera	*Gromphadorhina laevigata*	AY210820	AY210819
	Gromphadorhina laevigata	AY210820	AY210819
	Blattella germanica	DQ874116	AF005243
	Mantis religiosa	AY859586	AY859585
	Zootermopsis angusticollis	AY859615	AY859614

Table S4: Continued.

Taxon	Species	Genbank Acc. 18S	28S
	Ectobius lapponicus	DQ874125	EU426877
	Hierodula membranacea	AY491194	EU414720
Mantophasmatodea	*Mantophasma zephyra*	DQ874153	EU414719
	Tyrannophasma gladiator	AY521863	EU426875
Orthoptera	*Gomphocerinae sp.*	AY859547	AY859546
	Anacridium aegypticum	AY379759	EU414723
	Leptophyes punctatissima	AY521867	EU414721
	Pholidoptera griseoaptera	Z97587	EU414722
Phasmatodea	*Bacillus rossius*	AY121180	EU426879
	Carausius morosus	X89488	EU426878
Hemiptera	*Clastoptera obtusa*	AY744784	AF304569
	Pectinariophyes reticulata	AY744778	AF304570
	Prosapia bicincta	AY744789	AF304571
	Paracephaleus brunneus	AY498407	AF304626
	Cercopis vulnerata	AY744798	EU414724
	Harpocera thoracica	AY252388	EU414726
	Pyrrhocoris apterus	AY627318	EU414725
	Rhaphigaster nebulosa	X89495	EU426880
Coleoptera	*Tenebrio molitor*	X07801	AY210843
	Silpha obscura	AJ810737	EU426881
Hymenoptera	*Myrmecia croslandi*	AB121786	AB052895
	Vespula pensylvanica	AY859613	AY859612
	Nomada sp.	AY703484	EU414727
	Scolia sp.	EF012932	EU414728
	Tenthredinidae sp.	AF423781	EU414729
Lepidoptera	*Pieris napi*	AF423785	EU414731
Siphonaptera	*Ctenocephalides felis*	AF423914	EU414732
Mecoptera	*Merope tuber*	AF286287	DQ202351
	Boreus hyemalis	AF423882	EU426882
Trichoptera	*Oxyethira dualis*	AF423801	DQ202352
	Triaenodes sp.	AF286300	EU414730
Diptera	*Simulium sanctipauli*	AF403800	AF403820
	Acricotopus lucens	AJ586561	AJ586562
	Anopheles albimanus	L78065	L78065
	Aedes albopictus	X57172	L22060
	Chironomus tentans	X99212	X99212
	Drosophila melanogaster	M21017	M21017

Table S5: Species names and Genbank accession numbers to Anisoptera.

Taxon	Species	Genbank Acc. 12S+16S	28S
**Outgroups**			
Coenagrionidae	*Pyrrhosoma nymphula*		FJ596559
	Calopteryx splendens	EU477613	FJ596560
Epiophlebiidae	*Epiophlebia superstes*	EU477631	EU424328
**Anisoptera**			
Aeshnidae	*Caliaeschna microstigma*	EU477639	FJ596626
	Gomphaeshna furcillata	EU477638	FJ712314
	Boyeria irene	EU477640	FJ96627
	Brachytron pratense	EU477641	EU424323
	Gynacantha japonica	EU477644	FJ712316
	Anaciaeschna isoceles	EU477649	FJ596628
	Aeshna grandis	EU477645	FJ596630
	Aeshna juncea	EU477646	EU424324
	Aeshna mixta	EU477648	FJ712315
	Aeshna affinis	EU477647	FJ596629
	Anax parthenope	EU477651	FJ596631
	Anax imperator	EU477652	FJ596632
	Anax congoliath	EU477654	FJ596633
Austropetaliidae	*Phyllopetalia apicalis*	EU477637	FJ596639
Petaluridae	*Phenes raptor*	EU477632	FJ596624
	Uropetala carovei	EU477633	FJ596625
	Tachopteryx thoreyi	EU477636	FJ596623
	Tanypteryx pryeri	EU477635	FJ712330
Gomphidae	*Gomphus externus*	EU477655	FJ596612
	Gomphus exilis	EU477656	FJ596614
	Gomphus vulgatissimus	EU477660	FJ712320
	Progomphus obscurus	EU477677	EU424329
	Stylurus nagoyanus	EU477659	FJ596610
	Stylurus intricatus	EU477658	FJ712319
	Dromogomphus spinosus	EU477662	FJ596613
	Lanthus albistylus	EU477665	FJ596615
	Onychogomphus forcipatus	EU477669	FJ596619
	Onychogomphus forcipatus unguiculatus	EU477670	FJ596620
	Onychogomphus uncatus	EU477671	FJ712321
	Onychogomphus sp.	EU477672	FJ712322
	Ophiogomphus severus	EU477673	FJ596618
	Microgomphus chelifer	EU477678	FJ712323
	Stylogomphus shirizui	EU477679	FJ712325
	Neogomphus edenticulatus	EU477680	FJ712324

Table S5: Continued.

Taxon	Species	Genbank Acc. 12S+16S	28S
	Lindenia tetraphylla	EU477674	FJ596617
	Hagenius brevistylus	EU477667	FJ596621
	Leptogomphus sauteri	EU477681	FJ596609
	Asiagomphus melanops	EU477664	FJ596611
	Hemigomphus heterclytus	EU477683	FJ596622
Neopetaliidae	*Neopetalia punctata*	EU477684	FJ596636
Chlorogomphidae	*Chlorogomphus brunneus*	EU477691	FJ596635
Cordulegastridae	*Anotogaster sieboldi*	EU477686	FJ596638
	Cordulegaster boltoni	EU477688	FJ596634
	Cordulegaster picta	EU477685	FJ596637
	Cordulegaster maculata	EU477689	FJ712317
Macromiidae	*Phyllomacromia sp.*	JF912413	FJ596571
	Macromia amphigena	EU477699	FJ596570
	Macromia splendens	EU477696	FJ712329
Synthemistidae	*Synthemis eustalacta*	EU477693	FJ596574
	Eusythemis guttata	EU477692	FJ596573
Corduliidae s.l.	*Idomacromia proavita*	EU477694	FJ596572
	Oxygastra curtisii	EU477701	EU424325
	Gomphomacromia paradoxa	JF912412	FJ712318
	Epicordulia princeps	EU477710	FJ596565
	Cordulia aenea amurensis	EU477707	EU424326
	Epitheca bimaculata	EU477708	FJ596561
	Epitheca marginata	EU477711	FJ596561
	Hemicordulia mindana nipponica	EU477714	FJ596562
	Hemicordulia okinawensis	EU477715	FJ596563
	Somatochlora alpestris	EU477700	FJ596568
	Somatochlora flavomaculata	EU477704	EU424327
	Somatochlora metallica	EU477702	FJ596569
	Tetragoneura canis	EU477712	FJ596567
	Tetragoneura cynosura	EU477709	FJ596566
Libellulidae	*Acisoma panorpoides*	EU477753	FJ596592
	Acisoma trifida	EU477746	FJ596591
	Agrionoptera insignis	EU477738	FJ596599
	Allorhizucha preussi	EU477727	FJ596594
	Brachydiplax chalybea flavovittata	EU477717	FJ596585
	Celithemis elisa	EU477751	FJ596603
	Chalcostephia flavifrons	EU477716	FJ596584
	Cratilla metallica	EU477737	FJ596593
	Diastatops pullata	EU477757	FJ596579
	Hadrothemis infesta	EU477747	FJ596575
	Leucorrhinia pectoralis	EU477752	FJ596605
	Leucorrhinia dubia	EU477718	FJ596604
	Ladona fulva	EU477728	FJ596598
	Ladona depressa	EU477730	FJ596602
	Libellula quadrimaculata	EU477729	FJ712326
	Lyriothemis elegantissima	EU477735	FJ596600
	Macrodiplax cora	EU477722	FJ596588
	Nannophya pygmaea	EU477754	FJ596589
	Notiothemis robertsi	EU477725	FJ596583
	Orthemis discolor	EU477733	FJ596596
	Orthemis cultriformis	EU477734	FJ596597
	Orthetrum albistylum	EU477732	FJ596601
	Rhodothemis rufa	EU477744	FJ596590
	Rhyothemis variegata imperatrix	DQ021428	FJ596582
	Rhyothemis fuliginosa	EU477719	FJ712327
	Sympetrum danae	EU477740	EU424330
	Sympetrum vulgatum	EU477739	FJ596606
	Sympetrum striolatum	EU477741	FJ596608
	Sympetrum sanguineum	EU477743	FJ596607
	Sympetrum meridionale	EU477742	FJ712328
	Tetrathemis irregularis	EU477739	FJ596578
	Thermorthemis madagascariensis	EU477736	FJ596595
	Tholymis citrina	EU477721	FJ596577
	Tramea calverti	EU477750	FJ596586
	Trithemis africana	EU477756	FJ596580
	Trithemis aurora	EU477749	FJ596581
	Urothemis signata	EF640460	FJ596587
	Zygonyx torridus	EF640461	FJ596576

Table S6: Species names and Genbank accession numbers to Mammalia (Mitochondrion).

Taxon	Species	Genbank Acc.
Monotremata	*Ornithorhynchus anatinus*	NC_000891
	Tachyglossus aculeatus	NC_003321
	Zaglossus bruijni	NC_006364
Metatheria	*Caenolestes fuliginosus*	NC_005828
	Dactylopsila trivirgata	NC_008134
	Didelphis virginiana	NC_001610
	Distoechurus pennatus	NC_008145
	Dromiciops gliroides	NC_005826

Table S6: Continued.

Taxon	Species	Genbank Acc.
	Isoodon macrourus	NC_002746
	Macropus robustus	NC_001794
	Monodelphis domestica	NC_006299
	Notoryctes typhlops	NC_006522
	Phascolarctos cinereus	NC_008133
	Potorous tridactylus	NC_006524
	Rhyncholestes raphanurus	NC_005829
	Sminthopsis crassicaudata	NC_007631
	Trichosurus vulpecula	NC_003039
	Vombatus ursinus	NC_003322
Xenarthra	*Dasypus novemcinctus*	NC_001821
	Bradypus tridactylus	NC_006923
	Choloepus didactylus	NC_006924
	Tamandua tetradactyla	AJ421450
Afrotheria	*Chrysochloris asiatica*	NC_004920
	Dugong dugon	NC_003314
	Echinops telfairi	NC_002631
	Elephantulus sp.	NC_004921
	Elephas maximus	NC_005129
	Loxodonta africana	NC_000934
	Macroscelides proboscideus	NC_004026
	Mammut americanum	NC_009574
	Mammuthus primigenius	NC_007596
	Orycteropus afer	NC_002078
	Procavia capensis	NC_004919
Dermoptera	*Cynocephalus variegatus*	AJ428849
Lagomorpha	*Lepus europaeus*	NC_004028
	Ochotona collaris	NC_003033
	Ochotona princeps	NC_005358
	Oryctolagus cuniculus	NC_001913
Pholidota	*Manis tetradactyla*	NC_004027
Primates	*Cercopithecus aethiops*	AY863426
	Gorilla gorilla	NC_001645
	Homo sapiens	NC_001807
	Hylobates lar	NC_002082
	Macaca thibetana	NC_011519
	Pan paniscus	NC_001644
	Pan troglodytes	NC_001643
	Papio hamadryas	NC_001992
	Pongo pygmaeus	NC_001646
	Pygathrix nemaeus	NC_008220
	Aotus trivirgatus	AY250707
	Cebus albifrons	NC_002763
	Daubentonia madagascariensis	NC_010299
	Lemur catta	NC_004025
	Nycticebus coucang	NC_002765
	Propithecus verreauxi	AB286049
	Tarsius bancanus	NC_002811
Rodentia	*Anomalurus sp*	NC_009056
	Cavia porcellus	NC_000884
	Cricetulus griseus	NC_007936
	Jaculus jaculus	AJ416890
	Microtus rossiaemeridionalis	DQ015676.
	Mus musculus	NC_001569
	Myoxus glis	NC_001892
	Rattus norvegicus	AY172581
	Sciurus vulgaris	NC_002369
	Spalax ehrenbergi	AJ416891
	Thryonomys swinderianus	NC_002658
	Volemys kikuchii	AF348082
Scandentia	*Tupaia belangeri*	NC_002521
Carnivora	*Acinonyx jubatus*	NC_005212
	Ailuropoda melanoleuca	NC_009492
	Ailurus fulgens	NC_011124
	Arctocephalus foresteri	NC_004023
	Arctodus simus	NC_011116
	Canis familiaris	NC_002008
	Enhydra lutris	NC_009686
	Eumetopias jubatus	NC_004030
	Felis catus	NC_001700
	Halichoerus grypus	NC_001602
	Herpestes javanicus	NC_006835
	Meles meles	NC_011125
	Odobenus rosmarus	NC_004029
	Phoca vitulina	NC_001325
	Procyon lotor	NC_009126
	Spilogale putorius	NC_010497
	Tremarctos otus	NC_009969
	Ursus americanus	NC_003426
	Ursus arctos	NC_003427
	Ursus maritimus	NC_003428
	Vulpes vulpes	NC_008434
Cetartiodactyla	*Balaenoptera musculus*	NC_001601
	Balaenoptera physalus	NC_001321
	Berardius bairdii	NC_005274
	Bos taurus	NC_006853
	Bubalus bubalis	NC_006295

Table S6: Continued.

Taxon	Species	Genbank Acc.
	Camelus bactrianus	NC_009628
	Hippopotamus amphibius	NC_000889
	Lama pacos	NC_002504
	Lipotes vexillifer	NC_007629
	Muntiacus muntjak	NC_004563
	Ovis aries	NC_001941
	Physeter catodon	NC_002503
	Rangifer tarandus	NC_007703
	Sus scrofa	NC_000845
Perissodactyla	*Ceratotherium simum*	NC_001808
	Equus asinus	NC_001788
	Equus caballus	NC_001640
	Rhinoceros unicornis	NC_001779
	Tapirus terrestris	AJ428947
Chiroptera	*Artibeus jamaicensis*	NC_002009
	Chalinolobus tuberculatus	NC_002626
	Pipistrellus abramus	NC_005436
	Pteropus dasymallus	NC_002612
	Pteropus scapulatus	NC_002619
	Rhinolophus pumilus	NC_005434
	Rousettus aegyptiacus	NC_007393
Eulipotyphla	*Echinosorex gymnura*	NC_002808
	Erinaceus europaeus	NC_002080
	Hemiechinus auritus	NC_005033
	Hylomys suillus	NC_010298
	Crocidura russula	NC_006893
	Sorex unguiculatus	NC_005435
	Soriculus fumidus	AF348081
	Mogera wogura	NC_005035
	Talpa europaea	NC_002391
	Urotrichus talpoides	NC_005034

Table S7: Species names and Genbank accession numbers to Primates (Mitochondrion).

Taxon	Species	Genbank Acc.
**Outgroups**		
	Macropus robustus	NC_001794
	Balaenoptera musculus	NC_001601
	Equus caballus	NC_001640
	Ursus maritimus	NC_003428
**Primates**		
STREPSIRRHINI		
Lemuriformes	*Eulemur fulvus*	NC_012766
	Eulemur macaco	NC_012771
	Eulemur mongoz	NC_010300
	Lemur catta	NC_004025
	Propithecus coquereli	NC_011053
	Propithecus verreauxi	AB286049
	Varecia variegata	NC_012773
Lorisiformes	*Loris tardigradus*	NC_012763
	Otolemur crassicaudatus	NC_012762
	Nycticebus coucang	NC_002765
	Perodicticus potto	NC_012764
	Galago senegalensis	NC_012761
	Daubentonia madagascariensis	NC_010299
HAPLORRHINI		
Tarsiiformes	*Tarsius bancanus*	NC_002811
	Tarsius syrichta	NC_012774
Simiiformes		
Platyrrhini	*Aotus lemurinus*	FJ785421
	Callicebus donacophilus	FJ785423
	Cebus albifrons	NC_002763
	Saimiri sciureus	NC_012775
Catarrhini	*Ateles belzebuth*	FJ785422
	Cercopithecus aethiops	AY863426
	Chlorocebus aethiops	NC_007009
	Chlorocebus pygerythrus	NC_009747
	Chlorocebus tantalus	NC_009748
	Colobus guereza	NC_006901
	Gorilla gorilla	NC_011120
	Homo sapiens	NC_001807
	Homo sp.	NC_013993
	Hylobates agilis	NC_014042
	Hylobates lar	HQ622776
	Hylobates pileatus	NC_014045
	Macaca fascicularis	NC_012670
	Macaca mulatta	NC_005943
	Macaca sylvanus	NC_002764
	Macaca thibetana	NC_011519
	Nasalis larvatus	NC_008216
	Nomascus siki	NC_014051

Table S7: Continued.

Taxon	Species	Genbank Acc.
	Pan paniscus	NC_001644
	Pan troglodytes	NC_001643
	Papio hamadryas	NC_001992
	Pongo abelii	NC_002083
	Pongo pygmaeus	NC_001646
	Presbytis melalophos	NC_008217
	Procolobus badius	NC_008219
	Pygathrix nemaeus	NC_008220
	Pygathrix roxellana	DQ355297
	Semnopithecus entellus	NC_008215
	Symphalangus syndactylus	NC_014047
	Theropithecus gelada	FJ785426
	Trachypithecus obscurus	NC_006900

Table S8: Species names and Genbank accession numbers to Heterobranchia (28S).

Taxon	Species	Genbank Acc.
**Outgroups**		
Vetigastroda	*Lepetodrilus elevatus*	AY145413
Neritopsina	*Nerita funiculata*	DQ279976
Caenogastropoda	*Aperostoma palmeri*	DQ279983
	Juga acutifilosa	DQ256748
	Pleurocera canaliculatum	DQ256747
	Marstonia arga	DQ256749
	Bolinus brandaris	DQ279986
	Ilyanassa obsoleta	AY145411
**Heterobranchia**		
"Lower heterobranchs"		
	Graphis sp.	FJ917230
	Cima sp.	FJ917228
	Valvata piscinalis	FJ917224
	Cornirostra pellucida	FJ917225
	Boonea seminuda	AY145395
	Rissoella elongatospira	FJ917226
	Rictaxis punctocaelatus	FJ917243
	Striadorbis spiralis	DQ256746
	Glacidorbis rusticus	FJ917227
**Opisthobranchia**		
Cephalaspidea	*Haminoea solitaria*	AY145408
	Philine aperta	DQ279988
Notaspidea	*Umbraculum umbraculum*	FJ917246
Aplysiomorpha	*Aplysia californica*	AY026366
**Pulmonata**		
Amphiboloidea	*Phallomedusa solida*	DQ279991
	Phallomedusa solida #1	HQ156213
	Phallomedusa solida #2	HQ156220
	Salinator tecta #1	HQ156214
	Salinator tecta #2	HQ156215
Chilinoidea	*Latia neritoides*	FJ917245
Lymnaeoidea	*Austropeplea tomentosa*	HQ156217
	Fossaria obrussa	DQ256737
	Physa acuta	DQ256738
	Laevapex fuscus	DQ256734
	Glyptophysa gibbosa	DQ256736
	Micromenetus dilatatus	DQ256735
Siphonarioidea	*Siphonaria funiculata*	DQ256743
	Siphonaria pectinata	DQ256744
	Ophicardelus ornatus #1	DQ279994
	Ophicardelus ornatus #2	DQ256740
	Ophicardelus quoyi	DQ256739
Otinoidea	*Smeagol phillipensis*	FJ917229
Systellomatophora	*Onchidella patelloides*	HQ156218
	Onchidium verruculatum	DQ256742
	Onchidium damelii	HQ156219
	Vaginulus plebeius	DQ256745
Stylommatophora	*Deroceras reticulatum*	AY145404
	Arion silvaticus	AY145392
	Prietocella barbarae	HQ156216
	Helicodiscus parallelus	DQ256731
	Praticolella martensi	DQ256730
	Lamellaxis gracilis	DQ256733
	Mesomphix globosus	DQ256732
